# FLOT1 and EEF1D: ac4C-related genes bridging Alzheimer’s disease and sleep deprivation

**DOI:** 10.3389/fnagi.2026.1825164

**Published:** 2026-06-18

**Authors:** Beiyu Zhao, Rong Zhou, Peijie Liu, Qingli Li, Yulu Yan, Jing Du, Kaiyue Zhao, Jie Liu, Jin Wang, Qiumin Qu

**Affiliations:** Department of Neurology, The First Affiliated Hospital of Xi’an Jiaotong University, Xi’an, China

**Keywords:** ac4C-related genes, Alzheimer’s disease, EEF1D, FLOT1, sleep deprivation

## Abstract

**Background:**

Alzheimer’s disease (AD) and sleep deprivation (SD), two common conditions in the elderly, share complex molecular connections and may mutually influence each other’s pathogenesis. Current drugs only relieve symptoms with limited efficacy, making it urgent to explore the shared pathological mechanisms and potential intervention targets of the two conditions. This study used bioinformatics: first screening AD-related genes associated with SD and N4-acetylcytidine (ac4C) from relevant data; then identifying key genes via Mendelian randomization (MR) analysis and machine learning; finally screening AD-related key cells with single-cell RNA sequencing (scRNA-seq) data, to provide a basis for revealing the molecular and cellular regulatory mechanisms of AD-SD comorbidity.

**Methods:**

This study integrated bulk RNA sequencing (RNA-Seq) and scRNA-seq data from the Gene Expression Omnibus (GEO) database to identify AD-related key genes associated with SD and ac4C. Machine learning algorithms, including MR, were applied to screen these key genes. Additionally, gene set enrichment analysis (GSEA) was conducted to explore the pathways associated with the key genes, while ssGSEA was used to assess differences in immune cell infiltration. For the scRNA-seq data, key cells involved in AD pathology were further identified. Subsequently, the differential expression of the two key genes was validated using peripheral blood samples collected from AD and SD patients.

**Results:**

Through MR analysis, machine learning algorithms, and other analytical approaches, FLOT1 and EEF1D were identified as key genes. GSEA revealed that these key genes were enriched in multiple pathways, including the lysosome pathway, chemokine signaling pathway, and leukocyte transendothelial migration. Immune cell infiltration analysis suggested that myeloid-derived suppressor cells (MDSCs) might serve as key immune cells. Additionally, scRNA-seq analysis identified microglia, CD4 + T cells, CD8 + T cells, and natural killer (NK) cells as key cell types involved in AD pathogenesis. Critically, these key genes were successfully validated in peripheral blood samples from AD and SD patients, aligning with the above analysis.

**Conclusion:**

Overall, FLOT1 and EEF1D were identified as key genes associated with SD and ac4C in AD. This finding provided new grounds for the clinical diagnosis and treatment of AD.

## 1 Introduction

Alzheimer’s disease (AD), the most prevalent form of dementia, is fundamentally characterized by progressive cognitive decline and neuropsychiatric symptoms. Pathologically, it is defined by the deposition of *β*-amyloid (Aβ) plaques and the formation of neurofibrillary tangles due to hyperphosphorylated tau protein in the brain. The global prevalence of AD continues to rise, posing a severe threat to the health and quality of life of the elderly population (Khan et al., 2020; Twarowski and Herbet, 2023). Beyond the well-established amyloid and tau pathology, emerging research has increasingly recognized the complex interplay between sleep disturbances and AD pathogenesis. Accumulating evidence indicates that chronic sleep deprivation (SD) is associated with disrupted expression of circadian clock genes and altered neuropathological features in AD (Niu et al., 2022). Notably, clinical studies suggest that sleep disturbances and cognitive deficits in AD often co-occur and mutually exacerbate, with both tending to worsen during disease progression and cognitive decline (Wang and Holtzman, 2020; Tu et al., 2025). The complex relationship between sleep and AD pathology is further supported by animal studies, which demonstrate that SD is associated with increased microglial reactivity and Aβ deposition in a TREM2-dependent manner (Parhizkar et al., 2023). Currently available clinical treatments for AD offer only symptomatic relief and modest slowing of cognitive deterioration, without substantially halting or reversing disease progression. For managing SD in AD patients, benzodiazepines or non-benzodiazepine sedative-hypnotics are commonly prescribed. Although effective for short-term sleep improvement, long-term use carries risks of drug dependence and further cognitive impairment (McElroy et al., 2021; Fung et al., 2024). We systematically employed integrated bioinformatic approaches to identify ac4C-related genes and molecular pathways that may underlie the association between SD and AD, aiming to reveal shared pathological mechanisms linking these two conditions. Such research is crucial for developing novel intervention strategies that simultaneously improve sleep and slow AD progression—an essential direction for overcoming current therapeutic limitations.

To identify novel therapeutic targets, recent attention has turned to RNA epigenetic modifications, particularly N4-acetylcytidine (ac4C). This highly conserved modification, catalyzed by N-acetyltransferase 10 (NAT10), is widely distributed in transfer RNA (tRNA), ribosomal RNA (rRNA), and messenger RNA (mRNA) across eukaryotes, and functions primarily by regulating RNA splicing efficiency, intracellular transport, and translational stability, thereby playing critical roles in key physiological processes such as cell proliferation, differentiation, and stress responses (Wang et al., 2025). From a pathological perspective, emerging evidence has begun to reveal a potential link between ac4C modification and AD (Ji et al., 2025). In addition, the circadian rhythm—an endogenous 24-h regulatory system—relies fundamentally on the Clock/Bmal1–Per/Cry transcriptional negative feedback loop for its maintenance (Patke et al., 2020; Vasey et al., 2021). Circadian disruption is not only a significant contributor to SD but also exacerbates AD pathology by impairing Aβ clearance and promoting neuroinflammation. However, the specific mechanisms by which ac4C-related genes influence the pathogenesis of AD and SD remain poorly understood. Therefore, elucidating the regulatory role of ac4C modification in AD and SD is of considerable importance for facilitating breakthroughs in clinical therapies and promoting the development of more effective targeted drugs.

From a methodological standpoint, Mendelian randomization (MR) leverages genetic variants as instrumental variables, using summary data from genome-wide association studies (GWAS) to mitigate confounding and more accurately assess causal relationships between exposures and outcomes (Cao et al., 2022; Liu et al., 2024). Meanwhile, single-cell RNA sequencing (scRNA-seq) is a high-throughput transcriptomic technology capable of resolving gene expression profiles at the resolution of individual cells (Yu et al., 2023; Zhang X. et al., 2023). In contrast to conventional bulk RNA sequencing, which assesses gene expression at a multicellular level, scRNA-seq enables clear delineation of cellular heterogeneity and gene expression variability. It has been widely employed to decipher disease-specific transcriptomic landscapes. For instance, by analyzing scRNA-seq data from AD patients, researchers can identify aberrant cell-type-specific and region-specific modules at single-cell resolution and annotate transcriptomic differences associated with various pathological variables.

In the present study, we systematically employed integrated bioinformatic approaches to elucidate the molecular and cellular mechanisms by which ac4C-associated regulation mediates the bidirectional interplay between SD and AD pathogenesis. Given that NAT10 is widely expressed in the nervous system (Gao et al., 2024) and that ac4C modification patterns are highly conserved across different tissue types (Jiang et al., 2024), this study adopted the ac4C-related gene catalog, which was originally compiled from melanoma research, as a potential candidate molecular set for exploring epitranscriptomic dysregulation in neurodegenerative disorders. On this basis, we first identified ac4C-related differentially expressed genes from AD and SD datasets, and subsequently employed Mendelian randomization analysis to screen for genes with causal effects on AD risk. Key genes were subsequently identified through machine learning algorithms and expression validation. Further analyses included chromosomal localization, functional enrichment, and immune infiltration profiling of these candidate genes. Additionally, scRNA-seq was performed to identify critical cell subpopulations associated with key gene expression at single-cell resolution. Collectively, this multi-level investigation provides a comprehensive framework for understanding the interplay between SD and ac4C-related pathways in AD progression.

## 2 Materials and methods

### 2.1 Source of data

The Gene Expression Omnibus (GEO)^1^ database was utilized to obtain AD-related datasets. As a training set, the GSE63060 dataset (platform: GPL6947) comprised 145 blood samples from AD patients and 104 control samples (Sood et al., 2015). The GSE140829 dataset (platform: GPL15988) was utilized as a validation set, containing 204 blood samples from AD patients and 249 control samples (Wang et al., 2022). The GSE181279 dataset (platform: GPL24676) comprised 3 blood samples from AD patients and 2 control samples (Xu and Jia, 2021). Additionally, the GSE188545 dataset (platform: GPL24676) comprised 6 tissue samples from AD patients and 3 control samples (Zhang L. et al., 2023). SD-related datasets were also obtained from the GEO database. The GSE208668 dataset (platform: GPL10904), used as the training set, comprised 17 peripheral blood mononuclear cell (PBMC) samples from SD patients and 25 control samples (Piber et al., 2022). The GSE39445 dataset (platform: GPL15331) was utilized as a validation set, containing 217 blood samples from SD patients and 221 control samples (Moller-Levet et al., 2013). The genome-wide association study (GWAS) ID of AD was retrieved from the Integrative Epidemiology Unit (IEU) openGWAS database, and the GWAS ID ebi-a-GCST90027158 was selected for subsequent analyses. This dataset included 39,106 AD cases, 46,828 control samples, and 20,921,626 single-nucleotide polymorphisms (SNPs). Based on prior research (Liu et al., 2025), 2,118 ac4C-related genes (ac4CRGs) were retrieved (Supplementary Table 1). Drawing on previous research (Xiao et al., 2023), a total of 260 circadian rhythm-related genes (CRRGs) were obtained (Supplementary Table 2).

### 2.2 Differentially expressed genes (DEGs) identification

The “limma” package (v 3.44.3) was applied to perform differential expression analysis between AD and control groups in the GSE63060 dataset and between SD and control groups in the GSE208668 dataset, aiming to select DEGs (obtain DEGs-AD and DEGs-SD, respectively)(Ritchie et al., 2015). Genes were filtered using thresholds of *p* < 0.01 and |log_2_Fold Change (FC)| > 0.1 (Garreta et al., 2022; Tang et al., 2024). Volcano plots were generated applying the “ggplot” (v 3.5.1) package (Gustavsson et al., 2022), to visualize the expression patterns of DEGs in the GSE63060 and GSE208668 datasets.

### 2.3 Acquisition of candidate genes and their functional analysis

The “ggvenn” package (v 1.7.3) was used to intersect the up-regulated genes of both DEGs-AD and DEGs-SD, as well as their down-regulated genes (Gustavsson et al., 2022). The union of these 2 intersecting gene sets was then taken. Subsequently, the intersection between this union and the ac4CRGs was calculated to acquire the candidate genes. Aiming to explore the related biological functions and pathways, Gene Ontology (GO) and Kyoto Encyclopedia of Genes and Genomes (KEGG) enrichment analyses (*p* < 0.05) were performed on candidate genes applying the “clusterProfiler” package (v 4.8.3) (Wu et al., 2021). Specifically, GO biological functions were composed of cellular component (CC), molecular function (MF), and biological process (BP).

### 2.4 Mendelian randomization (MR) analysis

To investigate the causal relationship between candidate genes and AD, candidate genes were set as exposures and AD as the outcome. The TwoSampleMR package (v 0.6.14) was used to import exposures and screen instrumental variables (IVs) (Huang et al., 2023), with criteria: *p* < 5 × 10^−8^, clump = TRUE, *r*^2^ = 0.001, kb = 10, and *F* > 10. Only exposures with ≥3 SNP-IVs were retained. SNPs significantly associated with the outcome (*F*-statistics < 10) were excluded from the outcome GWAS data, followed by matching of exposure-SNP-outcome pairs. The TwoSampleMR package (v 0.6.14) was then applied to standardize effect alleles and sizes, and re-match exposures, IVs, and the outcome. Finally, the retained candidate genes were subjected to subsequent AD MR analysis.

Mendelian randomization analysis was performed using the mr function, with five algorithms incorporated: MR Egger, Weighted Median, Inverse Variance Weighted (IVW), Simple Mode, and Weighted Mode. The exposure–AD causal relationship was determined using IVW results. Scatter plots were generated via the mr_scatter_plot function, with emphasis on the IVW method. Forest plots of individual SNPs were constructed to visualize SNP-associated AD risk effects. Funnel plots were generated using the mr_funnel_plot function for heterogeneity assessment, where Q_pval > 0.05 indicated no significant heterogeneity and Q_pval < 0.05 indicated heterogeneity. In the horizontal pleiotropy test, a *p*-value > 0.05 indicated the absence of significant confounding effects from horizontal pleiotropy. Leave-one-out analysis was performed to evaluate the robustness of the overall study results. The Steiger directionality test was conducted to assess the direction of the causal association between each exposure and AD. Finally, results with a direction judgment of TRUE and a *p*-value < 0.05 were screened, and the corresponding genes were identified as candidate characteristic genes.

### 2.5 Identification of key genes

These candidate characteristic genes were uploaded to the Search Tool for the Retrieval of Interacting Genes/Proteins (STRING) database^2^, with the species specified as ‘*Homo sapiens*’ and an interaction score threshold set at >0.15. The PPI network was constructed using Cytoscape software (v 3.10.2) (Shannon et al., 2003). The MOCODE plugin analyzed the network, with Degree Cutoff set to 2 (Network Scoring) and Haircut algorithm selected in Cluster Finding (Node Score Cutoff = 0.2, K-Core = 2, Max. Depth = 100). Genes in the filtered subnetwork were defined as characteristic genes, to which machine-learning algorithms were applied. Specifically, analyses were conducted on training sets GSE63060 and GSE208668 to identify candidate key genes. In particular, least absolute shrinkage and selection operator (LASSO) regression analysis was conducted using the “glmnet” package (v 4.1.8), and the optimal model was obtained at the minimum lambda value (Friedman et al., 2010). Then, the candidate key genes were obtained by taking the intersection of genes obtained through LASSO analysis of the two data sets. In all samples from the training and validation sets of SD and AD, the Wilcoxon test was used to analyze candidate key gene expression differences between disease and control groups (*p* < 0.05). Key genes were ultimately identified as the intersection of genes passing expression validation for SD and AD, respectively.

### 2.6 Gene set enrichment analysis (GSEA)

Gene set enrichment analysis was implemented to comprehend the biological functions of key genes involved in the development of AD and SD. First, correlation coefficients were calculated using the “corrplot” package (v 0.92) for each key gene and all genes separately in all samples from GSE63060 and GSE208668, respectively (Zhang X. et al., 2023). Subsequently, the genes were sorted in descending order according to the correlation coefficients, thus generating a ranked list of genes corresponding to each key gene. Subsequently, GSEA was carried out. The reference gene set used for this analysis was “c2.cp.kegg.v2024.1.Hs.symbols.gmt” from the Molecular Signatures Database (MSigDB^3^) (*p* < 0.05, |Normalized Enrichment Score (NES)| > 1).

### 2.7 Key gene localization, correlation, and GeneMANIA analysis

The chromosomal distribution of key genes was visualized by applying the “RCircos” package (v 1.2.2) (Zhang et al., 2013). Subcellular localization of key genes was predicted via the mRNALocater database^4^. To explore interactions and functions of key genes with functionally similar genes, key genes were input into the GeneMANIA database^5^ for network construction and functional characterization. Correlations among key genes in AD and SD were investigated using their respective training sets. The expression levels of key genes in the two training sets were extracted, and the correlations among genes were calculated using the “corrplot” package (v 0.92) (|correlation coefficient (cor)| > 0.3, *p* < 0.05) (Zein and Akhtar, 2025). To explore the correlations between key genes and CRRGs in AD and SD datasets, the expression matrices of key genes were extracted based on the AD and SD training sets, and correlation analyses were performed using the “corrplot” package (v 0.92) (|cor| > 0.3, *p* < 0.05).

### 2.8 Immune cell infiltration analysis

Immune infiltration analysis was applied to probe the differences in the immune microenvironment between the AD and control groups of the training dataset GSE63060 and between the SD and control groups of the training dataset GSE208668. The infiltration scores for 28 immune cells were computed applying the “GSVA” package (v 1.50.0) (Hanzelmann et al., 2013; Jiang et al., 2022). Subsequently, the samples were filtered according to the confidence level of the samples, followed by comparing the differences in the infiltration scores of these immune cells between groups, applying the Wilcoxon test to obtain differential immune cells (DICs) (*p* < 0.05). Right after that, the “corrplot” package (v 0.92) was utilized to perform a correlation analysis, aiming to explore the potential associations both among DICs and between each key gene and DICs (|cor| > 0.3, *p* < 0.05).

### 2.9 scRNA-Seq data analysis

Low-quality cells were filtered from the AD single-cell datasets GSE181279 and GSE188545 using the “Seurat” package (v 5.0.1) with the following criteria: Excluding cells with the number of expressed genes (nFeature_RNA) counts < 200 or >4,000; Removing cells with the total number of gene expression counts (nCount_RNA) < 200 or >20,000; Excluding cells where the proportion of mitochondrial gene expression accounts for >20% of the total gene expression (Hao et al., 2021). The 2000 most variable genes were selected using the FindVariableFeatures function. Principal component analysis (PCA) dimensionality reduction was performed with the JackStrawPlot function to identify statistically significant principal components (*p* < 0.05). Unsupervised clustering of cells was conducted using the FindNeighbors and FindClusters functions, with clustering performed using the uniform manifold approximation and projection (UMAP) method (resolution = 0.2). Based on the clustering results, cell clusters were annotated with reference to the CellMarker 2.0 website (Xu and Jia, 2021). Key cells were identified according to the differential expression of key genes in cells. The “ReactomeGSA” package (v 1.12.0) was used to analyze the functional enrichment of key cells in single-cell datasets to explore their main involved biological functions (*p* < 0.05) (Griss et al., 2020).

### 2.10 Pseudo-time and cell communication analysis

In the GSE181279 and GSE188545 datasets, to understand the interactions between annotated cell clusters in AD and control groups, the “CellChat” package (v 2.2.0) was used to analyze the communication networks of different cell clusters, respectively (Fang et al., 2022). To infer gene regulatory trajectories of cell state transitions in the GSE181279 and GSE188545 datasets, key cell pseudo-time trajectories were constructed: dimensionality reduction and clustering were performed via the “Monocle” package (v 2.30.0) reduceDimension function (max_components = 2) (Cao et al., 2023); the orderCells function sorted key cells and assigned pseudo-time values based on key gene expression. Single-cell pseudo-time trajectories were built per key gene expression patterns, with the plot_genes_in_pseudotime function analyzing key gene expression during key cell differentiation. Additionally, key cell data was extracted for secondary clustering via the UMAP method (resolution = 0.2).

### 2.11 Transcription factor (TF) analysis

To comprehensively characterize TFs and their regulatory programs in each key cell, SCENIC analysis was performed via the Python software pySCENIC (v 0.12.1) using key cell data (Van de Sande et al., 2020). Finally, regulator activity in each key cell subpopulation was scored, and regulatory specificity scores (RSS) were generated to quantify TF specificity across different cell types.

### 2.12 Patient sample collection

Peripheral blood samples were collected from AD patients, SD patients and healthy controls using EDTA-K2 anticoagulant tubes, and stored at −80 °C until further test. The diagnosis of AD patients was based on the latest revised 2024 NIA-AA criteria (Jack et al., 2024), while SD patients were defined as those with a Pittsburgh Sleep Quality Index (PSQI) global score greater than 5 (Buysse et al., 1989), to ensure consistency with the diagnostic criteria applied in the bioinformatics analysis. Healthy controls were confirmed to have normal cognitive function, no significant sleep complaints, and no major neurological or psychiatric disorders. This study, which followed all protocols set forth in the Declaration of Helsinki, was approved by the Ethical Review Committee of the First Affiliated Hospital of Xi’an Jiaotong University (XJTU1AF2026LSYY-38), all participants provide written informed consent prior to participation.

### 2.13 Quantitative real-time PCR (qRT-PCR) analysis

Total RNA from whole blood was extracted using the RNAeasy™ Blood RNA Isolation Kit (Beyotime, China). RNA concentration and quality were measured using NanoDrop one Micro-Spectrophotometer (Thermo Fisher Scientific, USA). For cDNA synthesis, 500 ng of total RNA from each sample was reverse-transcribed using the Evo M-MLV RT Mix Kit (Accurate Biology, China). qRT-PCR was performed on CFX Connect™ Real-Time System (BIO-RAD, USA) using SYBR® Green Premix Pro Taq HS qPCR Kit (Accurate Biology, China). The relative expression level of target genes was calculated using the 2^−ΔΔCt^ method, with ACTB as an internal reference. The primer sequences employed in this study were as follows: EEF1D (Forward: 5′-GATTCCTCCTCCTTGGTCGC-3′; Reverse: 5′-CTTGTCTTCCCACACGGTCT-3′); FLOT1 (Forward: 5′-CCCTTCCCGAGACCCCA-3′; Reverse: 5′-CCTTGTTCTGCCCCTGGATT-3′); ACTB (Forward: 5′-TCACAATGTGGCCGAGGAC-3′; Reverse: 5′-TGGGGTGGCTTTTAGGATGG-3′).

### 2.14 Statistical analysis

Data were analyzed applying R software (v 4.3.3), and intergroup differences were evaluated via the Wilcoxon test or one-way ANOVA, with statistical significance defined as *p*-values < 0.05.

## 3 Results

### 3.1 Candidate gene identification and functional annotation analysis

**Figure 1:**
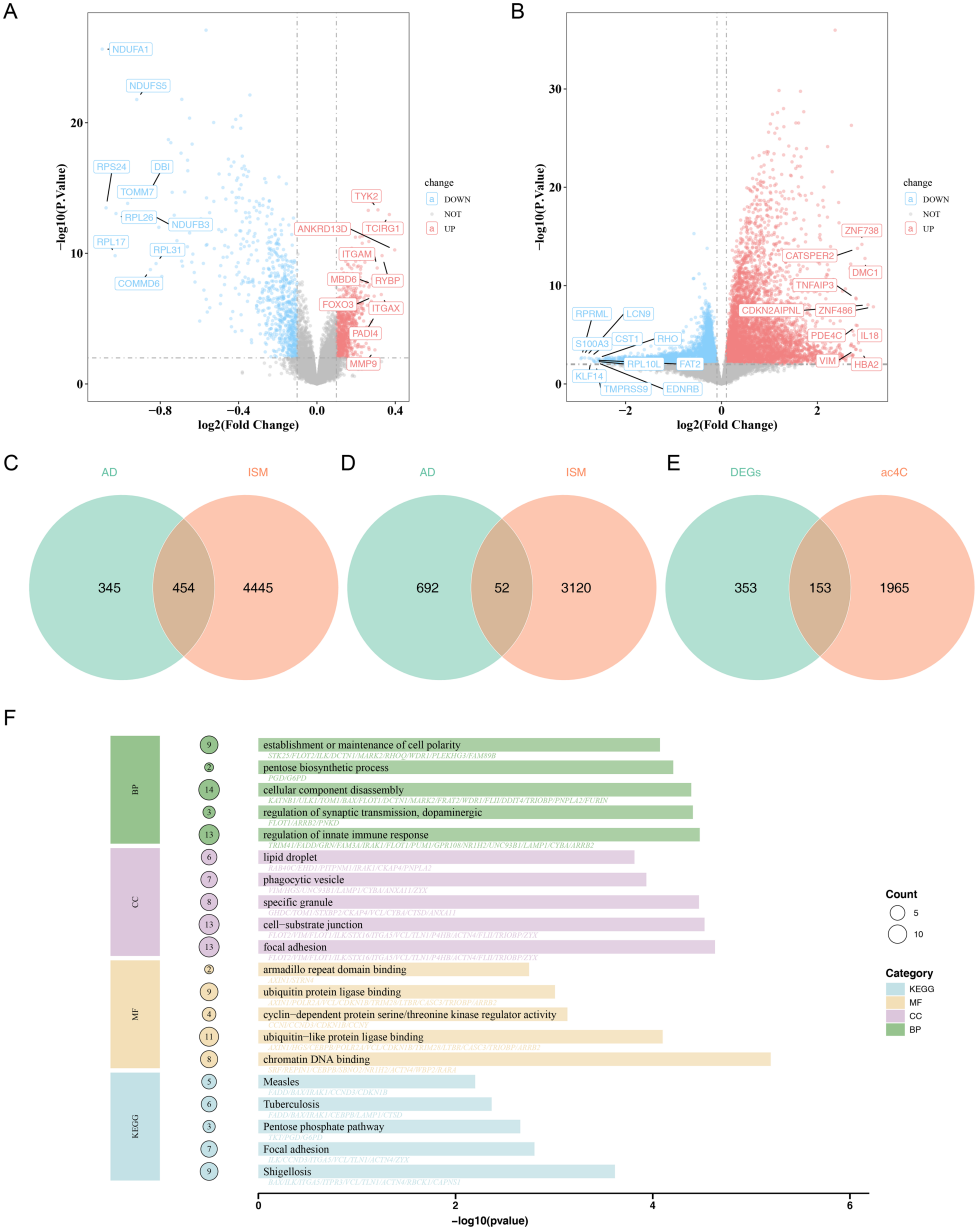
Analysis of DEGs in training sets. **(A)** Volcano plot in GSE63060 dataset; **(B)** Volcano plot in GSE208668 dataset; **(C)** This study intersected the up-regulated genes of both DEGs-AD and DEGs-SD; **(D)** This study intersected the down-regulated genes of both DEGs-AD and DEGs-SD; **(E)** Venn diagram showing candidate genes; **(F)** Functional enrichment analysis of candidate genes.

In total, 1,543 DEGs-AD were detected when comparing the AD and control groups in the GSE63060 dataset. Among these DEGs-AD, 799 genes exhibited significant up-regulation in the AD compared with the control groups, while 744 genes showed significant down-regulation (Figure 1A and Supplementary Table 3). Meanwhile, a total of 8,071 DEGs-SD were identified in the GSE208668 dataset through the comparison between the SD and control groups. Among these DEGs-SD, 4899 genes exhibited significant up-regulation in the SD compared with control groups, while 3,172 genes showed significant down-regulation (*p* < 0.01, |log_2_FC| > 0.1) (Figure 1B and Supplementary Table 4). This study intersected the up-regulated genes of both DEGs-AD and DEGs-SD, as well as their down-regulated genes. The union of these 2 intersecting gene sets was then derived to obtain 153 candidate genes (Figures 1C–E and Supplementary Table 5). Functional enrichment analysis was performed to characterize the biological functions and signaling pathways associated with the candidate genes (Figure 1F). The findings indicated that these candidate genes showed remarkable enrichment in 684 GO entries, which included 522 BPs, 92 CCs, and 70 MFs (*p* < 0.05) (Supplementary Table 6). These entries included regulation of innate immune response, cell-substrate junction, cyclin-dependent protein serine/threonine kinase regulator activity, and so on. Furthermore, these candidate genes demonstrated significant enrichment in 28 KEGG signaling pathways, including but not limited to focal adhesion (*p* < 0.05) (Supplementary Table 7).

### 3.2 Fifty-four candidate characteristic genes obtained by MR analysis

**Figure 2:**
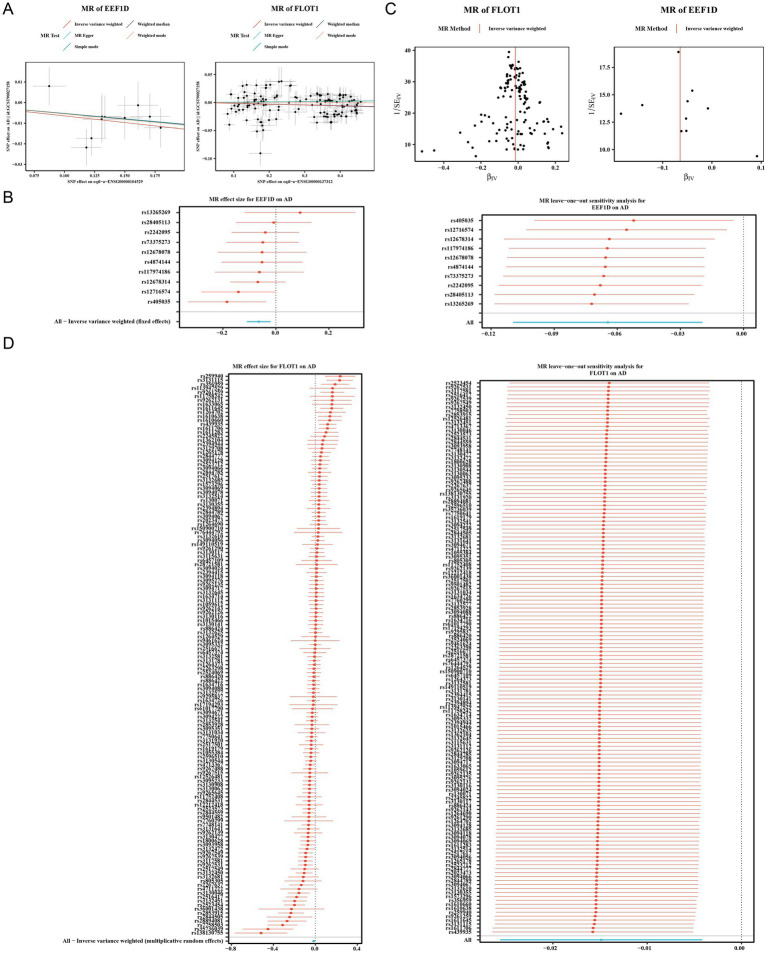
MR analysis. **(A)** Scatter plots of FLOT1 and EEF1D; **(B)** Forest plots of FLOT1 and EEF1D; **(C)** Funnel plots of FLOT1 and EEF1D; **(D)** Leave-one-out analysis forest plots of FLOT1 and EEF1D.

One hundred and fifty-three candidate genes were matched to 194 ENSEMBL IDs, and their 194 eQTL GWAS data were obtained from the Open GWAS database for subsequent analyses (Supplementary Table 8). Instrumental variable screening identified 76 exposure factors. Further IVW results showed that these 76 exposure factors had a significant causal relationship with AD (Supplementary Table 9), and subsequent correlation analyses were conducted with the FLOT1 and EEF1D genes as an example. In the correlation analysis between exposure factors and outcomes, the fitting lines of the five different algorithms for FLOT1 and EEF1D were all close to 0, suggesting that this study was less affected by confounding factors (Figure 2A). In the forest plot for EEF1D, all SNP effect sizes and the combined IVW effect were close to 0 or slightly below 0, indicating that EEF1D acts as a protective factor against AD. In the forest plot for FLOT1, most SNP effect sizes and the combined IVW effect were greater than 0, suggesting a positive correlation between FLOT1 and AD risk. (Figure 2B). In the MR randomness judgment analysis, the number of SNPs in the funnel plot of FLOT1 and EEF1D showed a roughly symmetric distribution on both sides (Figure 2C).

Further heterogeneity tests indicated that among the 76 exposure factors, 16 had Q_pval < 0.05 (indicating heterogeneity), and 60 had Q_pval > 0.05 (indicating no significant heterogeneity, and fixed-effect IVW was used for MR analysis) (Table 1). Horizontal pleiotropy analysis showed that 54 out of the 76 exposure factors had *p*-value> 0.05 and passed the horizontal pleiotropy test, indicating that these exposure factors were not disturbed by potential confounding factors, further ensuring the reliability and accuracy of the conclusions (Table 2). In the leave-one-out test, after sequentially removing each SNP of FLOT1 and EEF1D, the effect of the remaining SNPs on the outcome variable did not change significantly and all were on the left side of 0, and the other 53 genes also showed no significant changes, indicating that the MR analysis results were robust and reliable (Figure 2D). For genes with significant causal relationships in AD, Steiger directionality analysis was performed to verify the correctness of the direction (Table 3), and genes determined as TRUE with *p* < 0.05 were retained. The results showed that all exposure factors met the criteria, and finally, 54 genes passed the MR analysis as candidate characteristic genes (Supplementary Table 10).

**Table 1:** Results of the Mendelian randomization heterogeneity test.

id.exposure	Outcome	Method	Q_pval	SYMBOL
eqtl-a-ENSG00000010322	AD || id: GCST90027158	Inverse variance weighted	0.963839102	NISCH
eqtl-a-ENSG00000026025	AD || id: GCST90027158	Inverse variance weighted	0.585265198	VIM
eqtl-a-ENSG00000030582	AD || id: GCST90027158	Inverse variance weighted	8.44E-10	GRN
eqtl-a-ENSG00000031823	AD || id: GCST90027158	Inverse variance weighted	0.963575889	RANBP3
eqtl-a-ENSG00000035403	AD || id: GCST90027158	Inverse variance weighted	0.244254527	VCL
eqtl-a-ENSG00000051523	AD || id: GCST90027158	Inverse variance weighted	0.856765724	CYBA
eqtl-a-ENSG00000071127	AD || id: GCST90027158	Inverse variance weighted	0.932298904	WDR1
eqtl-a-ENSG00000071894	AD || id: GCST90027158	Inverse variance weighted	0.318872146	CPSF1
eqtl-a-ENSG00000072518	AD || id: GCST90027158	Inverse variance weighted	0.789129801	MARK2
eqtl-a-ENSG00000076924	AD || id: GCST90027158	Inverse variance weighted	0.997196477	XAB2

**Table 2:** Results of the Mendelian randomization horizontal pleiotropy test.

id.exposure	Outcome	pval	SYMBOL
eqtl-a-ENSG00000010322	AD || id: GCST90027158	0.009863945	NISCH
eqtl-a-ENSG00000026025	AD || id: GCST90027158	0.514911952	VIM
eqtl-a-ENSG00000030582	AD || id: GCST90027158	0.474388595	GRN
eqtl-a-ENSG00000031823	AD || id: GCST90027158	0.810226823	RANBP3
eqtl-a-ENSG00000035403	AD || id: GCST90027158	3.90E-07	VCL
eqtl-a-ENSG00000051523	AD || id: GCST90027158	0.98321187	CYBA
eqtl-a-ENSG00000071127	AD || id: GCST90027158	0.031236998	WDR1
eqtl-a-ENSG00000071894	AD || id: GCST90027158	0.000202969	CPSF1
eqtl-a-ENSG00000072518	AD || id: GCST90027158	0.022620025	MARK2
eqtl-a-ENSG00000076924	AD || id: GCST90027158	0.589098715	XAB2

**Table 3:** Steiger directionality test.

id.exposure	Outcome	Correct_causal_direction	Steiger_pval	SYMBOL
eqtl-a-ENSG00000015532	AD || id:ieu-b-4953	TRUE	2.71E-174	NISCH
eqtl-a-ENSG00000031691	AD || id:ieu-b-4953	TRUE	0	CENPQ
eqtl-a-ENSG00000047579	AD || id:ieu-b-4953	TRUE	0	DTNBP1
eqtl-a-ENSG00000064490	AD || id:ieu-b-4953	TRUE	0	RFXANK
eqtl-a-ENSG00000068489	AD || id:ieu-b-4953	TRUE	0	PRR11
eqtl-a-ENSG00000069020	AD || id:ieu-b-4953	TRUE	0	MAST4
eqtl-a-ENSG00000074201	AD || id:ieu-b-4953	TRUE	0	CLNS1A
eqtl-a-ENSG00000077238	AD || id:ieu-b-4953	TRUE	0	IL4R
eqtl-a-ENSG00000081692	AD || id:ieu-b-4953	TRUE	0	JMJD4
eqtl-a-ENSG00000088356	AD || id:ieu-b-4953	TRUE	1.52E-65	PDRG1

### 3.3 FLOT1 and EEF1D as key genes

PPI network analysis identified 1 discrete protein and obtained an interaction network of 53 proteins (Supplementary Figure 1A). Through MOCODE plug-in analysis, 11 characteristic genes were screened out (TRPC4AP, CLPTM1, RBCK1, RANBP3, POLG, ACTN4, FLOT1, RHOQ, EEF1D, PGD, TKT) (Supplementary Figures 1B–D). In the AD training set GSE63060, LASSO analysis showed that when lambda. Min = 0.04552, the regression coefficients of 7 genes (TRPC4AP, RBCK1, RANBP3, POLG, FLOT1, RHOQ, EEF1D) were not penalized to zero (Supplementary Figures 1E,F). In the SD training set GSE208668, LASSO analysis showed that when lambda. Min = 0.00015, the regression coefficients of 8 genes (TRPC4AP, CLPTM1, RANBP3, POLG, ACTN4, FLOT1, RHOQ, EEF1D) were not penalized to zero (Supplementary Figures 1G,H). The intersection of the above two LASSO analysis results yielded 6 candidate key genes (Supplementary Figure 1I).

Furthermore, in the AD training set GSE63060 and validation set GSE140829, TRPC4AP, FLOT1, and EEF1D showed significant expression differences between the AD group and the control group, with consistent expression trends (*p* < 0.05) (Supplementary Figures 2A,B). In the SD training set GSE208668 and validation set GSE39445, FLOT1 and EEF1D showed significant expression differences between the SD group and the control group, with consistent expression trends (*p* < 0.05) (Supplementary Figures 2C,D). In conclusion, FLOT1 and EEF1D were identified as key genes (Supplementary Figure 2E).

### 3.4 Key genes shared enriched signaling pathways

**Figure 3:**
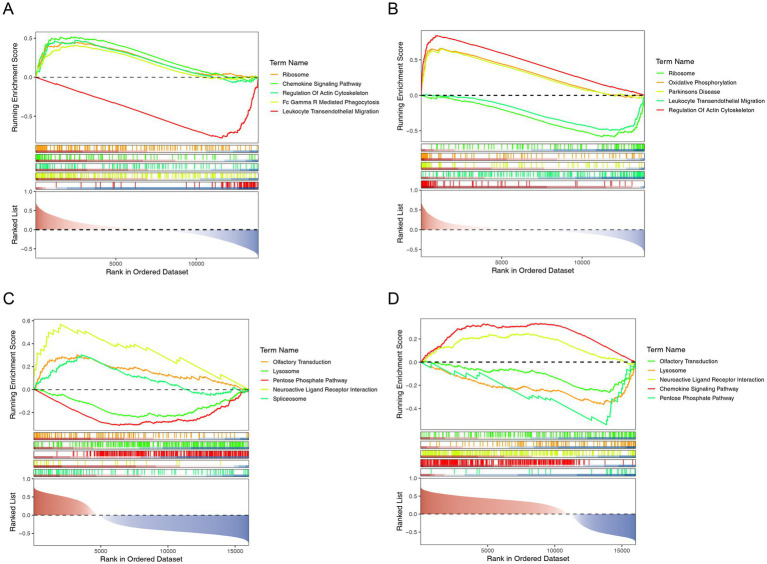
Key genes shared enriched signaling pathways. **(A)** FLOT1 in GSE63060; **(B)** EEF1D in GSE63060; **(C)** FLOT1 in GSE208668; **(D)** EEF1D in GSE208668.

The GSEA results for GSE63060 indicated that the top 5 significantly enriched pathways (*p* < 0.05, |NES| > 1) for the key genes were as follows. FLOT1 and EEF1D were enriched in 118 and 102 pathways, respectively (Figures 3A,B and Supplementary Tables 11, 12), including the chemokine signaling pathway and leukocyte transendothelial migration. For GSE208668, the top 5 significantly enriched pathways (*p* < 0.05, |NES| > 1) for the key genes included lysosome, neuroactive ligand receptor interaction, and chemokine signaling pathway. Specifically, FLOT1 and EEF1D were enriched in 99 and 88 pathways, respectively (Figures 3C,D and Supplementary Tables 13, 14).

### 3.5 Key gene localization, correlation, and GeneMANIA analysis

Chromosomal localization showed that FLOT1 was located on chromosome 6 and EEF1D on chromosome 8 (Supplementary Figure 3A); subcellular localization analysis indicated that the mRNAs of FLOT1 and EEF1D were mainly distributed in the cytoplasm (Supplementary Figure 3B). In addition, results of GeneMANIA functional enrichment analysis showed that the two key genes (FLOT1 and EEF1D) were primarily involved in processes related to regulation of protein stability and translational regulator activity (Supplementary Figure 3C).

In the AD training set, the two key genes showed a significant negative correlation (cor = −0.32, *p* < 0.05) (Supplementary Figure 3D); in the SD training set, they also exhibited a significant negative correlation (cor = −0.64, *p* < 0.05) (Supplementary Figure 3E). In the AD training set, among the correlations between CRRGs and key genes, the strongest negative correlation was between RPS27A and FLOT1, and the strongest positive correlation was between CSNK1D and FLOT1 (Supplementary Figure 4). In the SD training set, the strongest negative correlation was between PROK2 and EEF1D, and the strongest positive correlation was between CSNK1D and FLOT1 (|cor| > 0.3, *p* < 0.05) (Supplementary Figure 5).

### 3.6 Immune microenvironment profiling

**Figure 4:**
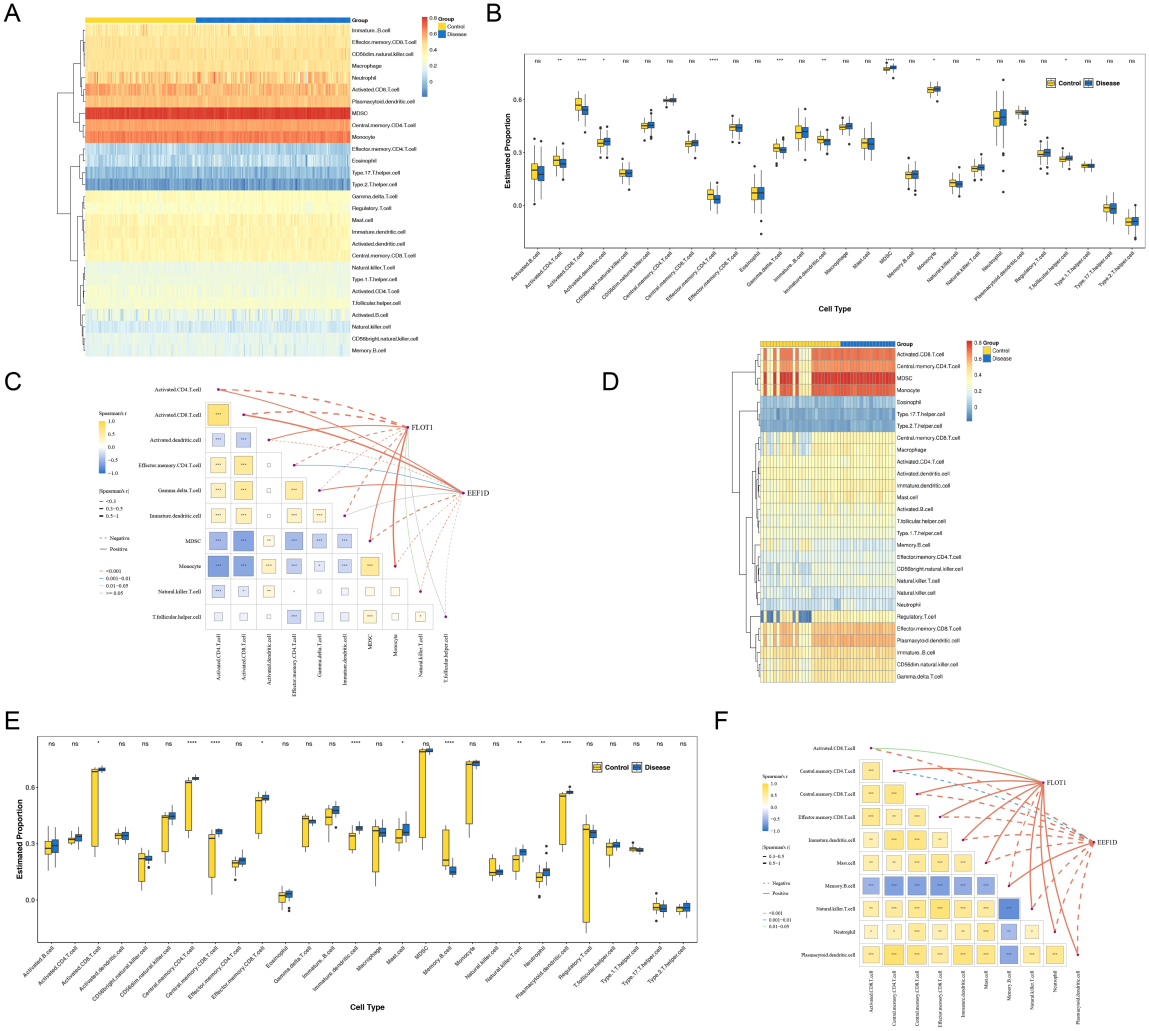
Key genes were implicated in the immune microenvironment of AD/SD. **(A,D)** Heatmaps of different cell contents inferred by ssGSEA; **(B,E)** Expression profiles of immune-infiltrating cells; **(C,F)** Correlation analysis.

In the GSE63060 dataset, both the AD group and the control group showed a high proportion of myeloid-derived suppressor cells (MDSCs) (Figure 4A). A total of 10 statistically significant immune cell differential interactions (DICs, *p* < 0.05) were identified (Figure 4B), including MDSCs, monocytes, natural killer T (NK T) cells, etc. Among these, for immune cell pairs, the strongest positive correlation was observed between activated CD4 T cells and activated CD8 T cells (cor = 0.68, *p* < 0.01), and the strongest negative correlation was found between monocytes and activated CD4 T cells (cor = −0.67, *p* < 0.01). For key genes, EEF1D exhibited the strongest negative correlation with activated CD8 T cells (cor = −0.55, *p* < 0.01), while FLOT1 showed the strongest positive correlation with activated CD8 T cells (cor = 0.61, *p* < 0.01) (Figure 4C).

In the GSE208668 dataset, both the SD group and the control group had a high proportion of MDSCs (Figure 4D). Ten immune cell DICs (*p* < 0.05) were analyzed (Figure 4E), including central memory CD4 T cells, mast cells, etc. Among these, for immune cell pairs, the strongest significant positive correlation was between central memory CD4 T cells and plasmacytoid dendritic cells (cor = 0.74, *p* < 0.01), and the strongest negative correlation was between NKT cells and memory B cells (cor = −0.79, *p* < 0.01). For key genes, EEF1D displayed the strongest negative correlation with mast cells (cor = −0.76, *p* < 0.01), while FLOT1 had the strongest positive correlation with plasmacytoid dendritic cells (cor = 0.78, *p* < 0.01) (Figure 4F).

### 3.7 Cell annotation and key cell identification

**Figure 5:**
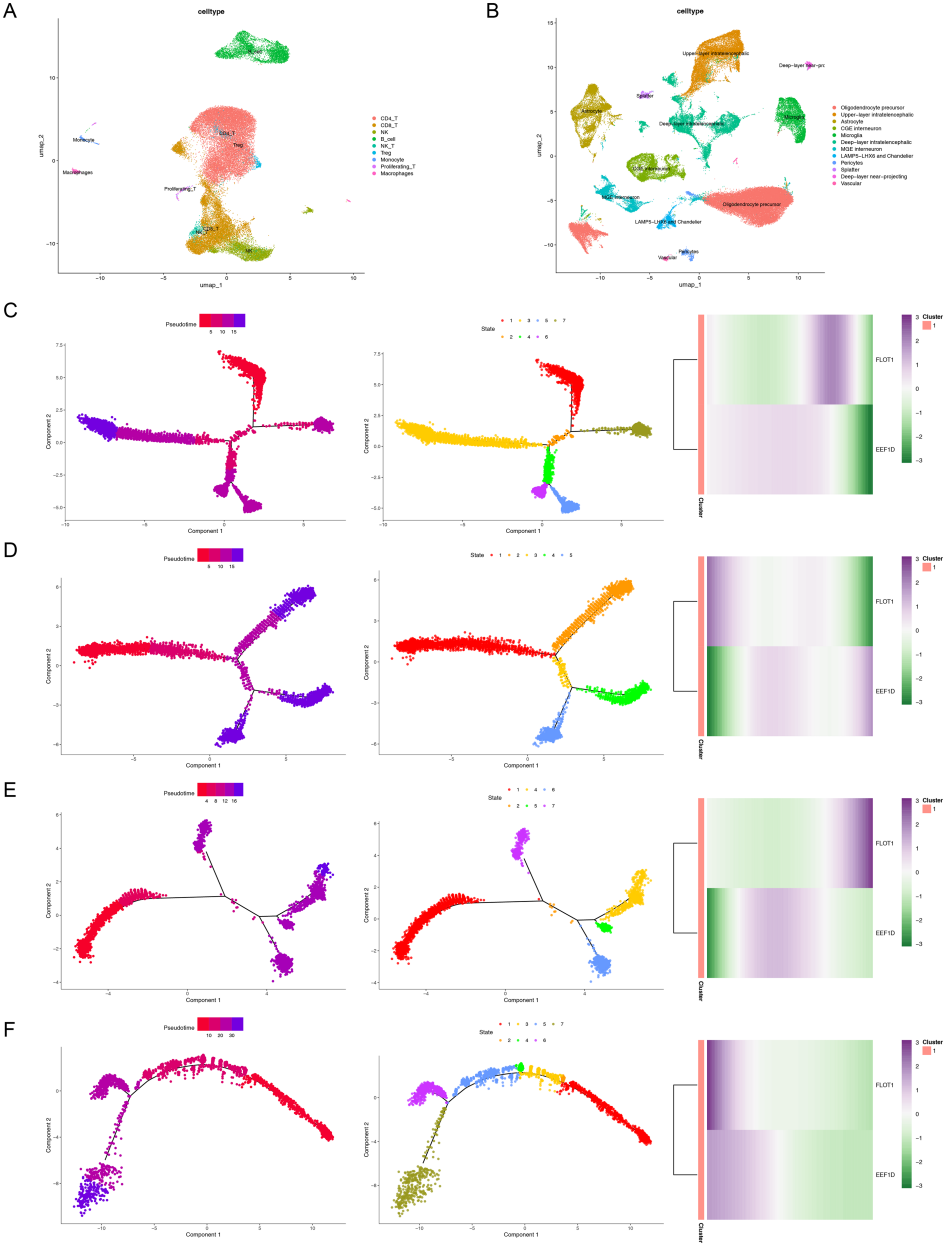
Cell annotation and pseudo-time analysis. **(A)** GSE181279 cell annotation, **(B)** GSE188545 cell annotation, **(C)** Pseudo-time series analysis and key gene expression of CD4 T cells, **(D)** Pseudo-time series analysis and key gene expression of CD8 T cells, **(E)** Pseudo-time series analysis and key gene expression of NK cells, **(F)** Pseudo-time series analysis and key gene expression of microglia.

After data quality control, the GSE181279 dataset yielded 36,720 cells and 17,727 genes (Supplementary Figures 6A,B). The top 30 principal components (parameter: dims = 30) were selected for subsequent analysis and clustered into 14 cell clusters (Supplementary Figures 6C–F), which were further annotated into 9 cell types (Figure 5A and Supplementary Figure 6G). In addition, T cells accounted for the highest proportion in both the control group and all experimental groups, while B cells had a relatively higher proportion in the AD group (Supplementary Figure 6H). The two candidate key genes were differentially expressed in CD4 T, CD8 T, and NK cells, thus identifying these three cell types as key cells (*p* < 0.05) (Supplementary Figure 6I). Cell pathway enrichment analysis showed that key cells were enriched in pathways including selenocysteine synthesis and eukaryotic translation termination (Supplementary Figure 6J). After data quality control, the GSE188545 dataset yielded 80,078 cells and 28,314 genes (Supplementary Figures 7A,B). The top 30 principal components (parameters: dims = 30) were selected for analysis, clustered into 20 cell clusters and annotated into 12 cell types (Figure 5B and Supplementary Figures 7C–G). Furthermore, oligodendrocyte precursor cells accounted for the highest proportion in the control group and all experimental groups (Supplementary Figure 7H). The two key genes were differentially expressed in microglia (*p* < 0.05), leading to the identification of microglia as key cells (Supplementary Figure 7I). Cell pathway enrichment analysis indicated that these 12 cell types were co-enriched in pathways such as mitochondrial transcription termination (Supplementary Figure 7J).

### 3.8 Pseudo-time analysis

Secondary clustering analysis of key cells in the GSE181279 dataset showed that CD4 T cells were finally divided into 4 clusters, CD8 T cells into 8 clusters, and NK cells into 4 clusters (Supplementary Figures 8A–C). CD4 T cells were projected onto a developmental trajectory with 1 root and 6 branches, which was divided into 7 stages according to developmental status: Stage 1 was the early differentiation stage, Stages 2 and 4 were the early-middle differentiation stages, Stages 5, 6, and 7 were the middle-late differentiation stages, and Stage 3 was the late differentiation stage. During its development, FLOT1 showed a relatively high expression level in the middle-late stages, while EEF1D was highly expressed from the early to the middle-late stages (Figure 5C). CD8 T cells were projected onto a developmental trajectory with 1 root and 4 branches, which was divided into 5 stages (Stages, corresponding to different colors) based on developmental status: Stage 1 was the early differentiation stage, Stage 3 was the early-middle differentiation stage, Stage 2 was the middle-late differentiation stage, and Stages 4 and 5 were the late differentiation stages. During its development, the expression level of FLOT1 decreased gradually from the early to the middle-late stages; EEF1D was highly expressed in the early-middle stages, then decreased, and increased again in the late stages (Figure 5D). NK cells were projected onto a developmental trajectory with 1 root and 6 branches, which was divided into 7 stages (Stages, corresponding to different colors) according to developmental status: Stage 1 was the early differentiation stage, Stages 3 and 7 were the early-middle differentiation stages, Stages 5 and 6 were the middle-late differentiation stages, and Stage 4 was the late differentiation stage. During its development, FLOT1 was highly expressed in the late stages, while EEF1D was highly expressed from the early-middle to the middle-late stages (Figure 5E). Secondary clustering analysis of key cells in the GSE188545 dataset showed that microglia were finally divided into 6 clusters (Supplementary Figure 8D). They were projected onto a developmental trajectory with 1 root and 6 branches, which was divided into 7 stages (Stages, corresponding to different colors) according to developmental status: Stage 1 was the early differentiation stage, Stages 2, 3, 4, and 5 were the early-middle differentiation stages, Stage 6 was the middle-late differentiation stage, and Stage 7 was the late differentiation stage. During the development of microglia, both FLOT1 and EEF1D were highly expressed in the early stage, and their expression levels gradually decreased afterward (Figure 5F).

### 3.9 Cell communication analysis

**Figure 6:**
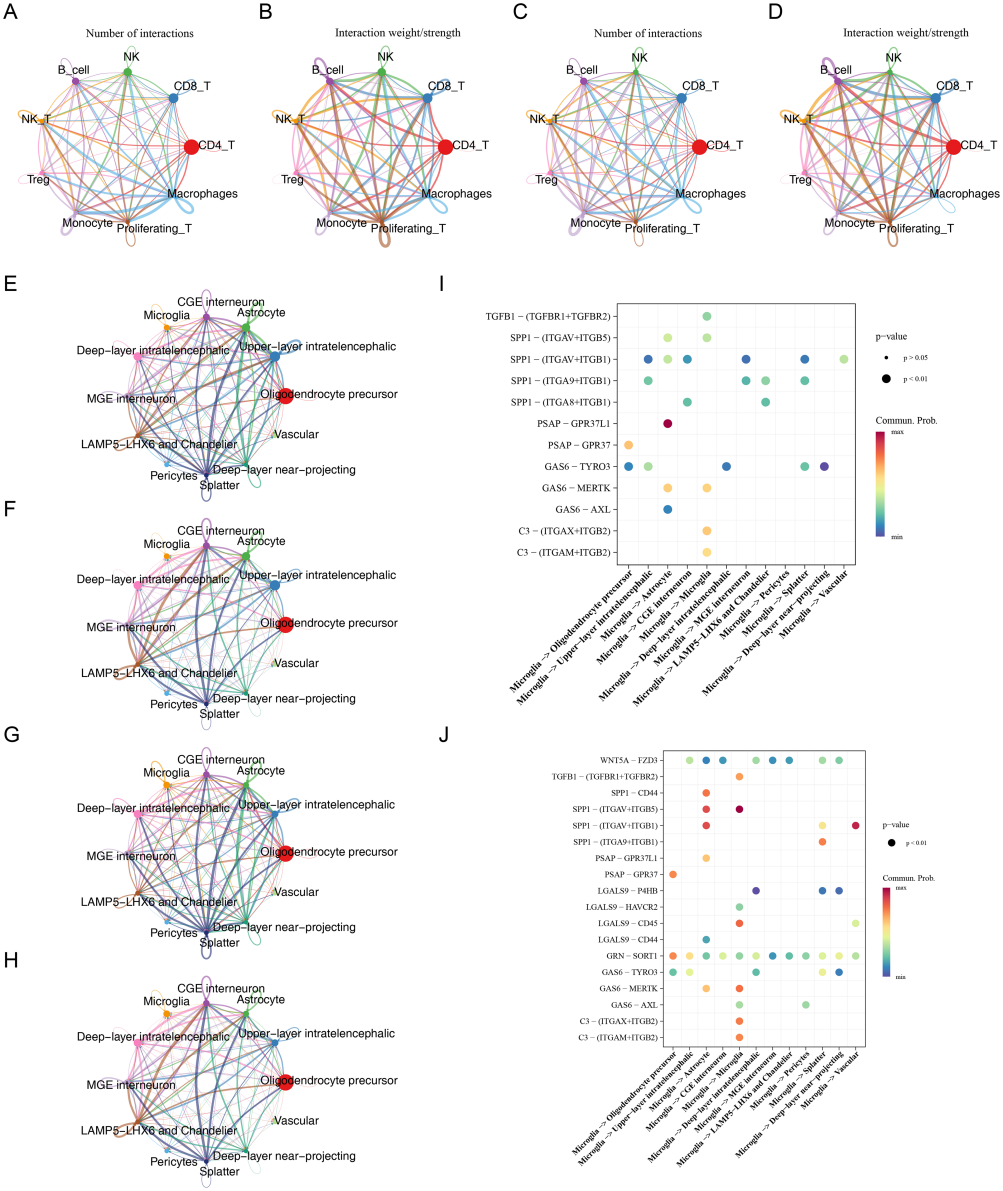
Cell communication analysis. **(A)** Number of cell communication events in the control group, GSE181279 dataset, **(B)** Intensity of cell communication in the control group, GSE181279 dataset, **(C)** Number of cell communication events in the AD group, GSE181279 dataset, **(D)** Intensity of cell communication in the AD group, GSE181279 dataset, **(E)** Number of cell communication events in the control group, GSE188545 dataset, **(F)** Intensity of cell communication in the control group, GSE188545 dataset, **(G)** Number of cell communication events in the AD group, GSE188545 dataset, **(H)** Intensity of cell communication in the AD group, GSE188545 dataset, **(I)** Ligand-receptor communication probability analysis between cells in the control group, GSE188545 dataset, **(J)** Ligand-receptor communication probability analysis between cells in the AD group, GSE188545 dataset.

In the GSE181279 dataset, cell communication analysis of the control group demonstrated that CD4 T cells sent and received fewer signals with low intensity; CD8 T cells sent fewer signals but received more, also with low intensity; NK cells sent and received more signals with high intensity (Figures 6A,B). In the AD group, CD4 T cells, CD8 T cells, and NK cells all exhibited the characteristic of “fewer signals sent, more signals received, and low intensity” (Figures 6C,D). In the control group, CD4 T cells, CD8 T cells, and NK cells all had the strongest communication association with B cells (Supplementary Figures 9A–C). In the AD group, the communication probability between the MIF molecules on the surface of these three cell types (CD4 T cells, CD8 T cells, and NK cells) and the CD74 and CXCR4 molecules on the surface of B cells was the highest (Supplementary Figures 9D–F). In addition, cell communication analysis of the GSE188545 dataset demonstrated that microglia in the control group sent and received fewer signals with low intensity (Figures 6E,F). In the AD group, they sent and received more signals, but the intensity remained low (Figures 6G,H). In the control group (Figure 6I), among the communications where microglia acted as ligand cells and other cells as receptor cells, the communication probability between the PSAP molecule of microglia and the GPR37L1 molecule of astrocytes was the highest; the analysis of microglial ligand-receptor interactions in the AD group showed that the communication probability between the SPP1 molecule of microglia and its own ITGAV and ITGB5 molecules was the highest (Figure 6J).

### 3.10 Evaluate the relationship between key genes and TF regulation in key cells

**Figure 7:**
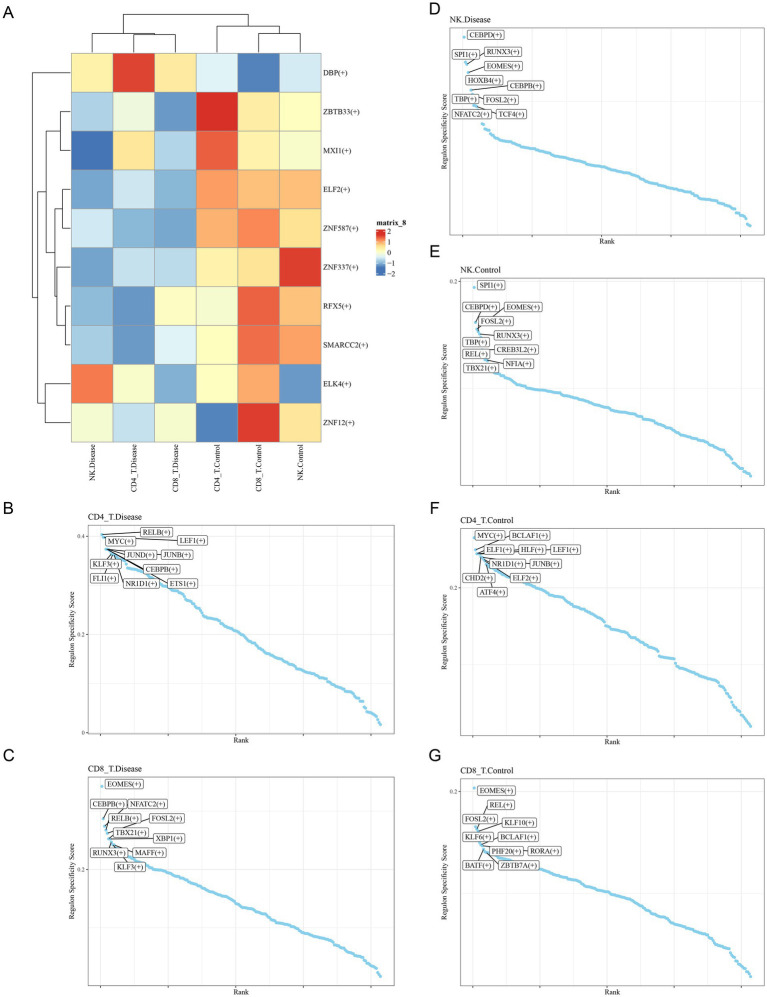
Evaluate the relationship between key genes and TF regulation in key cells. **(A)** Heatmap of AUC scores for the top 10 TFs ranked by AUC score; **(B–G)** RSS score plots of each TF in each cell subset under different disease status groups of key subsets. The closer to the left, the higher the ranking.

Evaluation and analysis of the regulatory relationships between key genes and TFs in key cells identified 207 TFs in the GSE181279 dataset (Supplementary Table 15). By calculating the total under the curve (AUC) values of each subset of key cells (CD4T, CD8T, NK cells), the top 10 TFs were selected after sorting by AUC values in descending order (Figure 7A). It was found that the AUC scores of these top 10 TFs had significant differences among groups with different AD statuses in the same cell type. The results of RSS scores showed that the top 10 high-scoring TFs in the six subsets of key cells were different, which might suggest that different subset cells had distinct transcriptional regulatory states (Figures 7B–G). Evaluation and analysis of the regulatory relationships between key genes and TFs in key cells identified 194 TFs in the GSE188545 dataset (Supplementary Table 16). The AUC score heatmap of TFs related to key genes showed that TBP and XBP1 had significantly different activities in the disease group and the control group. After selecting the top 10 TFs by sorting AUC values in descending order, it was found that their AUC scores had obvious differences among groups with different disease statuses in the same cell type (Supplementary Figures 10A,B). The results of RSS scores under different disease statuses showed that the top 10 high-scoring TFs in the key cell subsets were different, which might suggest that different subset cells had distinct transcriptional regulatory states (Supplementary Figures 10C,D).

### 3.11 Target genes validation using qRT-PCR

**Figure 8:**
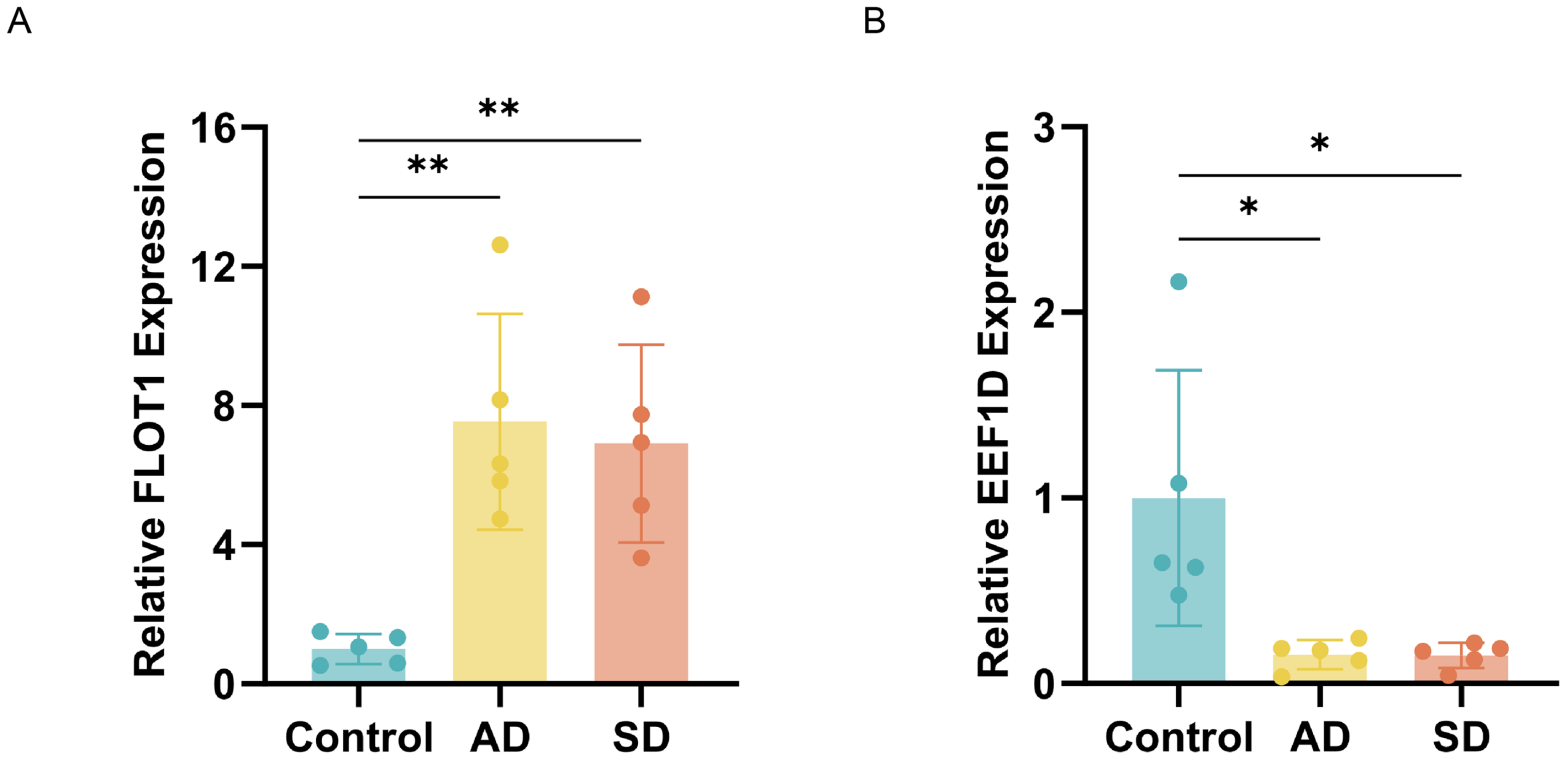
qRT-PCR of FLOT1 and EEF1D. **(A)** The expression of FLOT1 was significantly elevated in the blood of both AD and SD patients; **(B)** The expression of EEF1D was decreased in the blood of both AD and SD patients compared with control. * for *P* < 0.05 and ** for *P* < 0.01.

To verify the expression trends of EEF1D and FLOT1 in AD and SD, we collected peripheral blood samples from patients and healthy controls. The groups showed good comparability in basic demographic characteristics and lifestyle factors. Clinical information indicated that there were no significant differences among the control, AD, and SD groups in terms of age, sex, comorbidities, or lifestyle factors (all *p* > 0.05), whereas significant differences were observed only in disease-related clinical assessment indicators (MMSE, MoCA, and PSQI) (*p* < 0.05) (Table 4). On this basis, qRT-PCR was employed to detect the expression levels. The results revealed that, compared with the healthy control group, FLOT1 expression in the blood of AD and SD patients was significantly increased, whereas EEF1D expression was significantly decreased (Figure 8).

**Table 4:** Clinical information form.

Characteristics	Control (*n* = 5)	AD (*n* = 5)	SD (*n* = 5)	*p-*value
Age, years*	62 (13)	65 (9)	66 (13)	0.992
Male (n, %)	3 (60.0)	3 (60.0)	3 (60.0)	1.000
MMSE*	30 (1.50)	17 (6.00)	30 (2.00)	**0.007**
MoCA*	30 (1.50)	11 (8.50)	30 (2.00)	**0.005**
PSQI*	4 (1.50)	4 (1.50)	10 (7.00)	**0.009**
Self-reported comorbidities, *n* (%)				
Hypertension	1 (20.0)	1 (20.0)	2 (40.0)	1.000
Diabetes	0 (0)	0 (0)	0 (0)	/
Stoke	0 (0)	0 (0)	0 (0)	/
Coronary heart disease	0 (0)	1 (20.0)	1 (20.0)	1.000
Hypothyroidism	0 (0)	0 (0)	0 (0)	/
Lifestyle, *n* (%)				
Smoking	0 (0)	0 (0)	1 (20.0)	1.000
Drinking	0 (0)	0 (0)	1 (20.0)	1.000

^*^Continuous variables are presented as median (interquartile range). P-value were calculated using the Mann–Whitney *U* test. Bold values indicate statistical significance at *p* < 0.05. MMSE, minimum Mental State Examination; MoCA, Montreal Cognitive Assessment; PSQI, Pittsburgh sleep quality index.

## 4 Discussion

The present study integrated bioinformatic approaches, including Mendelian randomization, machine learning, and single-cell RNA sequencing, to systematically identify FLOT1 and EEF1D as key genes connecting SD and ac4C-associated regulation in the AD-SD bidirectional pathology. Further functional analysis revealed that these genes are significantly enriched in the lysosome and chemokine signaling pathways, suggesting their potential involvement in AD pathology through the regulation of intracellular degradation and immune signal transduction. Consistent with this, immune infiltration analysis demonstrated an increased abundance of MDSC under both AD and SD conditions, indicating that immune-mediated mechanisms may constitute a common pathological foundation for the convergence of the two disorders.

FLOT1 encodes flotillin-1, a member of the flotillin family predominantly localized in lipid raft structures of the plasma membrane. Physiologically, FLOT1 participates in lipid raft-mediated cellular processes, including signal transduction, vesicular trafficking, endocytosis, and neuronal homeostasis. Accumulating evidence supports its relevance to AD pathogenesis. Dysregulated FLOT1 expression—either overexpression or underexpression—may disrupt neural homeostasis and contribute to multiple neurological disorders, including AD, Parkinson’s disease, and major depressive disorder (Zhan et al., 2023). Consistent with its pathological significance, flotillin family proteins have been proposed as promising biomarkers for AD (Angelopoulou et al., 2020). Critically, flotillin-1 was observed to abnormally accumulate within lysosomes of neurofibrillary tangle-bearing neurons in AD (Girardot et al., 2003). Such lysosomal sequestration of FLOT1 likely impairs lysosomal function, thereby hampering the clearance of pathological proteins such as tau and exacerbating neurofibrillary tangle formation. Additionally, this mislocalization disrupts lipid raft-mediated signaling and trafficking, ultimately compromising neuronal survival and synaptic integrity—core processes underlying cognitive decline.

Furthermore, FLOT1 may potentially be involved in the pathophysiology of SD. The enrichment of FLOT1 in the lysosome pathway implies that its aberrant localization and function may disrupt the metabolism and recycling of key sleep-regulating molecules, such as enzymes involved in melatonin synthesis or receptors governing circadian rhythms, thereby impairing precise regulation of the sleep–wake cycle. Furthermore, FLOT1 dysfunction may contribute to both neuronal impairment in AD and sleep–wake dysregulation, potentially through shared mechanisms involving lysosomal dysfunction and altered lipid raft signaling. The involvement of FLOT1 in both conditions suggests it may represent a molecular link underlying their frequent comorbidity. Therefore, aberrant subcellular localization of FLOT1 may represent a key molecular node connecting the pathogenesis of both conditions.

Beyond FLOT1, our analysis identified EEF1D as another key gene mechanistically linking AD and SD pathogenesis. EEF1D encodes eukaryotic translation elongation factor 1 delta (eEF1δ), an essential subunit of the eEF1 complex that plays critical roles in energy metabolism and maintenance of translational complex stability. Accumulating evidence has implicated EEF1D in the pathogenesis of neurological disorders. Genetic analysis in a Chinese family identified compound heterozygous variants in EEF1D as causative for autosomal recessive intellectual disability, characterized by cognitive developmental delay and adaptive functioning deficits (Zhang et al., 2024). Further supporting this association, biallelic loss-of-function mutations in EEF1D were demonstrated to disrupt the heat shock response pathway—a crucial mechanism for neuronal protection through regulated stress protein synthesis—thereby contributing to the pathogenesis of autosomal recessive intellectual disability (Ugur Iseri et al., 2019). Expanding the clinical spectrum of EEF1D-related neurodevelopmental impairments, a subsequent study revealed that biallelic variants in its guanine exchange domain likewise lead to neurodevelopmental abnormalities manifesting as speech delay and motor dysfunction (Averdunk et al., 2023).

Based on the above findings, we hypothesize that EEF1D dysfunction contributes to AD pathology through two mechanisms: impaired translational efficiency and dysregulated heat shock response, thereby reducing the ability of neurons to resist Aβ toxicity and tau aggregation, and exacerbating stress-induced damage and cognitive decline. Notably, SD also involves disturbances in translational regulation and heat shock response. Previous studies showed that SD induced the unfolded protein response in the cerebral cortex, leading to significant changes in the phosphorylation levels of translation factors including elongation factors, and reduced global protein translation activity (Gronli et al., 2012). In addition, SD broadly induces the expression of heat shock protein family mRNAs in multiple brain regions, which may represent a neuroprotective response to prolonged wakefulness (Terao et al., 2003). Given that EEF1D possesses both classical translation elongation functions and transcriptional regulatory functions in the heat shock response, its reduced expression may, on one hand, exacerbate translation inhibition induced by SD and interfere with the synthesis of sleep-regulatory proteins; on the other hand, it may affect the transcriptional regulation of heat shock proteins, thereby weakening the adaptive capacity of neurons to the stress of sleep loss. In summary, EEF1D dysfunction may participate in both AD neurodegeneration and the pathophysiology of SD by simultaneously impairing neuroprotective translation and stress adaptation mechanisms, providing a molecular explanation for the frequent comorbidity of these two conditions.

Having identified key genes and their associated signaling pathways, we next investigated the immune microenvironment to determine whether immune dysregulation represents a shared mechanism linking AD and SD. Our immune infiltration analysis revealed a significantly elevated relative abundance of myeloid-derived suppressor cells (MDSCs) in both AD and SD groups, suggesting their potential role as key immunomodulatory hubs linking immune dysregulation in these two conditions. MDSCs may contribute to shared pathological progression by remodeling the local immune microenvironment in both disorders. The scRNA-seq analysis further identified multiple immune cell populations—including CD4 + T cells, CD8 + T cells, NK cells, and microglia—as critically involved in AD pathogenesis. These findings imply that AD development involves not only aberrant microglial activation within the central nervous system but also coordinated responses from peripheral immune cells. Although CD4 + T, CD8 + T, NK cells, and MDSCs differ in origin, phenotype, and functional specialization, they collectively regulate immune homeostasis and play essential roles in infection clearance, tumor surveillance, inflammatory responses, and disease pathogenesis. Accumulating evidence supports their involvement in AD. For instance, CD4 + T cells exhibit context-dependent effects in AD; pro-inflammatory subsets such as Th1 and Th17 produce key inflammatory cytokines that compromise blood–brain barrier integrity and promote astrocyte activation, thereby facilitating Aβ production and deposition (Kubick et al., 2020). Similarly, CD8 + effector memory CD45RA + (TEMRA) cells—identified as an immune signature cell type in AD—correlate negatively with cognitive performance (Gate et al., 2020).

The scRNA-seq further revealed enhanced T-cell receptor signaling in CD8 + TEMRA cells from AD patients, and clonally expanded CD8 + TEMRA populations were detected in the cerebrospinal fluid, suggesting their active involvement in AD-related neuroinflammation. Moreover, AD brains upregulate various chemokines and cytokines that recruit and expand MDSCs, fostering an immunosuppressive microenvironment that impedes Aβ clearance and neural repair (Salminen et al., 2018). Additionally, NK cells exhibit transcriptomic alterations in AD, with studies demonstrating connections between NK cell dysfunction, aging, and AD pathogenesis at single-cell resolution (Qi et al., 2022; Li et al., 2024). Additionally, microglia-driven neuroinflammation is well established as a central mechanism in AD progression (Wang et al., 2023). Collectively, the coordinated involvement of both central microglia and diverse peripheral immune cell populations in AD pathogenesis supports a central-peripheral immune synergy model of AD immunopathology. In this framework, microglia mediate direct neural damage through local inflammation, while peripheral immune cells—including pro-inflammatory CD4 + T subsets, CD8 + TEMRA cells, dysregulated NK cells, and MDSCs—contribute to disease progression via barrier disruption, exacerbated inflammation, and impaired repair. The shared elevation of MDSCs in AD and SD further implies that common immune dysregulation mechanisms may underlie both conditions. Thus, MDSCs may act as a critical immunological nexus, with central microglial activation and peripheral immune disturbances jointly driving AD pathology and its comorbidity with SD.

This study has several limitations. Although the qRT-PCR results from peripheral blood align with bioinformatics trends, the validation cohort was small and consisted of retrospectively collected samples from a single center. Consequently, the differential expression of FLOT1 and EEF1D in AD and SD requires confirmation in larger, multicenter, prospective cohorts. Second, the association of key genes with ac4C modification was indirectly inferred from published gene sets and lacks direct acRIP-sequencing evidence from AD or SD tissues. Additionally, all analyses relied on transcriptomic and single-cell data from public databases, which offer limited resolution regarding ethnicity, disease subtype, and clinical stratification, potentially limiting the generalizability of the findings. Finally, although bioinformatics analyses provide potential research directions and statistical support for the comorbidity, the precise regulatory network linking FLOT1 and EEF1D to AD–SD comorbidity remains to be elucidated. Future work will focus on: systematically evaluating the biomarker potential of these genes in multicenter clinical cohorts; directly validating ac4C modification status and functional consequences through acRIP-sequencing combined with NAT10 perturbation experiments; and dissecting the *in vivo* molecular pathways through which these key genes regulate neuroinflammation and synaptic homeostasis using comorbidity animal models and organoid platforms. These efforts will establish an experimental foundation for therapeutic strategies targeting the epitranscriptome.

In summary, this study identified 54 genes with causal links to AD and significant associations with SD through Mendelian randomization and differential expression analysis. Machine learning approaches prioritized FLOT1 and EEF1D as key regulators, with enrichment analyses implicating chemokine signaling and lysosome pathways as shared mechanisms. Immune profiling revealed elevated myeloid-derived suppressor cells as a potential immunological bridge between these conditions. The scRNA-seq further demonstrated the involvement of both central microglial activation and peripheral immune responses in AD pathogenesis. While limited by sample size and methodological constraints, these findings provide novel insights into the molecular interplay between AD and SD.

## Data Availability

The datasets presented in this study can be found in online repositories. The names of the repository/repositories and accession number(s) can be found in the article/Supplementary material.

## Footnotes

1
http://www.ncbi.nlm.nih.gov/geo/

2
http://www.string-db.org/

3 https://www.gsea-msigdb.org/gsea/msigdb

4
http://bio-bigdata.cn/mRNALocater/

5
http://www.genemania.org

## Supplementary material

SUPPLEMENTARY FIGURE 1 Screen candidate key genes. **(A)** PPI network; **(B)** Cluster 1; **(C)** Cluster 2; **(D)** Cluster 3; **(E,G)** represent the error plots of Lasso cross-validation for AD and SD, respectively. A positive coefficient indicates that high expression of the gene increases the risk of the outcome, while a negative coefficient indicates that high expression of the gene reduces the risk of the outcome. Curves (in different colors): Changes in the regression coefficients of genes under different λ values; as λ increases, some curves gradually decrease to 0, indicating that the corresponding gene is eliminated by LASSO; **(F,H)** represent the gene coefficient plots of Lasso for AD and SD, respectively. Lines and labels in different colors: Each point represents a model corresponding to a λ value; colors and labels indicate the number of variables retained under that λ; **(I)** Venn diagram.

SUPPLEMENTARY FIGURE 2 Screen key genes via expression level evaluation. **(A)** GSE63060; **(B)** GSE140829; **(C)** GSE208668; **(D)** GSE39445. * indicated *P* < 0.05, ** indicated *P* < 0.01, *** indicated *P* < 0.001, **** indicated *P* < 0.0001; **(E)** Venn diagram.

SUPPLEMENTARY FIGURE 3 Key gene localization, correlation, and GeneMANIA analysis. **(A)** Chromosomal localization; **(B)** Subcellular localization; **(C)** GeneMANIA analysis; **(D)** Correlation analysis of key genes in the AD training set; **(E)** Correlation analysis of key genes in the SD training set.

SUPPLEMENTARY FIGURE 4 Correlation analysis between key genes and CRRGs in the AD training set.

SUPPLEMENTARY FIGURE 5 Correlation analysis between key genes and CRRGs in the SD training set.

SUPPLEMENTARY FIGURE 6 GSE181279 dataset: cell annotation and key cell identification. **(A)** Before quality control, **(B)** after quality control, **(C)** screening of highly variable genes, **(D)** principal component scatter plot, **(E)** principal component scree plot, **(F)** UMAP dimensionality reduction and clustering plot of cells, **(G)** bubble plot of marker gene expression across different cell clusters, **(H)** cell proportion plot, **(I)** expression of key genes in different cell types, **(J)** enrichment analysis of key cell populations. (* indicated *P* < 0.05, ** indicated *P* < 0.01, *** indicated *P* < 0.001, **** indicated *P* < 0.0001).

SUPPLEMENTARY FIGURE 7 GSE188545 dataset: cell annotation and key cell identification. **(A)** Before quality control, **(B)** after quality control, **(C)** screening of highly variable genes, **(D)** principal component scatter plot, **(E)** principal component scree plot, **(F)** UMAP dimensionality reduction and clustering plot of cells, **(G)** bubble plot of marker gene expression across different cell clusters, **(H)** cell proportion plot, **(I)** expression of key genes in different cell types, **(J)** enrichment analysis of key cell populations. (* indicated *P* < 0.05, ** indicated *P* < 0.01, *** indicated *P* < 0.001, **** indicated *P* < 0.0001).

SUPPLEMENTARY FIGURE 8 The secondary clustering analysis of key cells. **(A)** CD4 T cells, **(B)** CD8 T cells, **(C)** NK cells, **(D)** Microglia.

SUPPLEMENTARY FIGURE 9 Analysis of intercellular ligand-receptor communication probability (GSE181279). **(A–C)** Analysis of ligand-receptor communication probabilities between cells in the Control group; **(D–F)** Analysis of ligand-receptor communication probabilities between cells in the AD group.

SUPPLEMENTARY FIGURE 10 Evaluate the relationship between key genes and TF regulation in key cells. **(A)** Heatmap of TF AUC scores related to key genes; **(B)** Heatmap of AUC scores for the top 10 TFs ranked by AUC score in microglia under different disease statuses; **(C,D)** RSS score plots of each TF in each subset of cells under different disease status groups of key subsets. The closer to the left, the higher the ranking.

SUPPLEMENTARY TABLE 1 List of ac4C-related genes.

SUPPLEMENTARY TABLE 2 List of circadian rhythm-related genes.

SUPPLEMENTARY TABLE 3 List of DEGs-AD.

SUPPLEMENTARY TABLE 4 List of DEGs-SD.

SUPPLEMENTARY TABLE 5 List of candidate genes.

SUPPLEMENTARY TABLE 6 GO enrichment.

SUPPLEMENTARY TABLE 7 KEGG enrichment.

SUPPLEMENTARY TABLE 8 One hundred ninety-four eQTL GWAS data.

SUPPLEMENTARY TABLE 9 Further IVW results showed that these 76 exposure factors had a significant causal relationship with AD.

SUPPLEMENTARY TABLE 10 List of candidate characteristic genes.

SUPPLEMENTARY TABLE 11 GSEA analysis for FLOT1 in GSE63060.

SUPPLEMENTARY TABLE 12 GSEA analysis for EEF1D in GSE63060.

SUPPLEMENTARY TABLE 13 GSEA analysis for FLOT1 in GSE208668.

SUPPLEMENTARY TABLE 14 GSEA analysis for EEF1D in GSE208668.

SUPPLEMENTARY TABLE 15 Two hundred seven lists of TFs.

SUPPLEMENTARY TABLE 16 One hundred ninety-four lists of TFs.

## Glossary

ac4C: N4-acetylcytidine
AD: Alzheimer’s disease
Aβ: *β*-amyloid
BP: biological process
CC: cellular component
CRRGs: circadian rhythm-related genes
DEGs: Differentially expressed genes
DICs: differential immune cells
FC: Fold Change
GEO: Gene Expression Omnibus
GO: Gene Ontology
GSEA: gene set enrichment analysis
GWAS: genome-wide association studies
IEU: Integrative Epidemiology Unit
IVW: Inverse Variance Weighted
KEGG: Kyoto Encyclopedia of Genes and Genomes
LASSO: least absolute shrinkage and selection operator
MDSCs: myeloid-derived suppressor cells
MF: molecular function
MR: Mendelian randomization
NAT10: N-acetyltransferase 10
NK: natural killer
PBMC: peripheral blood mononuclear cell
PCA: Principal component analysis
PSQI: Pittsburgh Sleep Quality Index
qRT-PCR: Quantitative real-time PCR
RNA-Seq: RNA sequencing
RSS: regulatory specificity scores
scRNA-seq: single-cell RNA sequencing
SD: sleep deprivation
SNPs: single-nucleotide polymorphisms
STRING: Search Tool for the Retrieval of Interacting Genes/Proteins
TF: Transcription factor
UMAP: uniform manifold approximation and projection
