# Supplementary figures and images for: FLOT1 and EEF1D: ac4C-related genes bridging Alzheimer’s disease and sleep deprivation
Source: Front Aging Neurosci. 2026 Jun 18;18:1825164. doi: 10.3389/fnagi.2026.1825164 (PMC13323225; doi:10.3389/fnagi.2026.1825164)

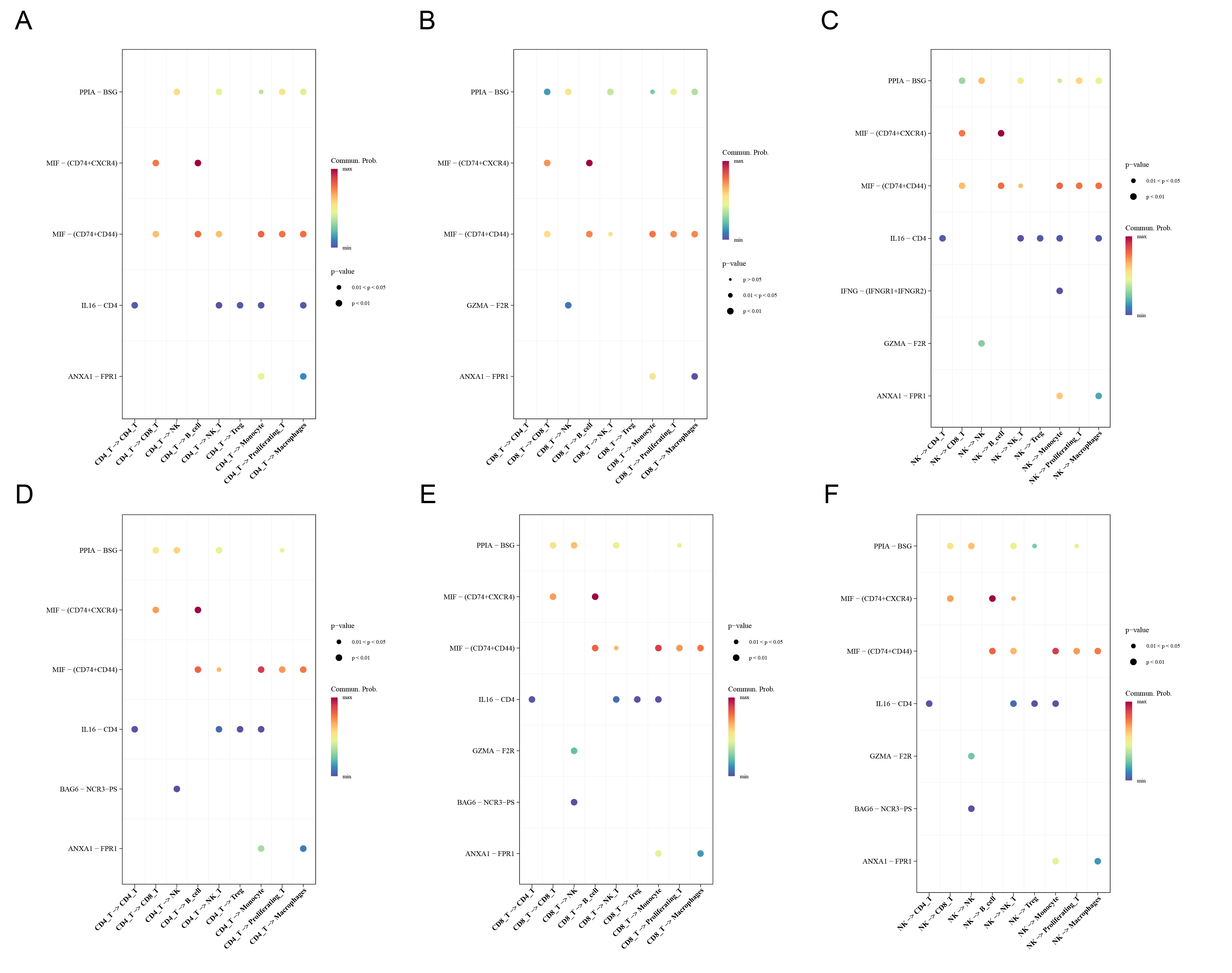

Supplement: SUPPLEMENTARY FIGURE 1 — Screen candidate key genes. (A) PPI network; (B) Cluster 1; (C) Cluster 2; (D) Cluster 3; (E,G) represent the error plots of Lasso cross-validation for AD and SD, respectively. A positive coefficient indicates that high expression of the gene increases the risk of the outcome, while a negative coefficient indicates that high expression of the gene reduces the risk of the outcome. Curves (in different colors): Changes in the regression coefficients of genes under different λ values; as λ increases, some curves gradually decrease to 0, indicating that the corresponding gene is eliminated by LASSO; (F,H) represent the gene coefficient plots of Lasso for AD and SD, respectively. Lines and labels in different colors: Each point represents a model corresponding to a λ value; colors and labels indicate the number of variables retained under that λ; (I) Venn diagram. [file Data_sheet_1.zip › Supplementary material/Supplementary Figure. 9.tif]

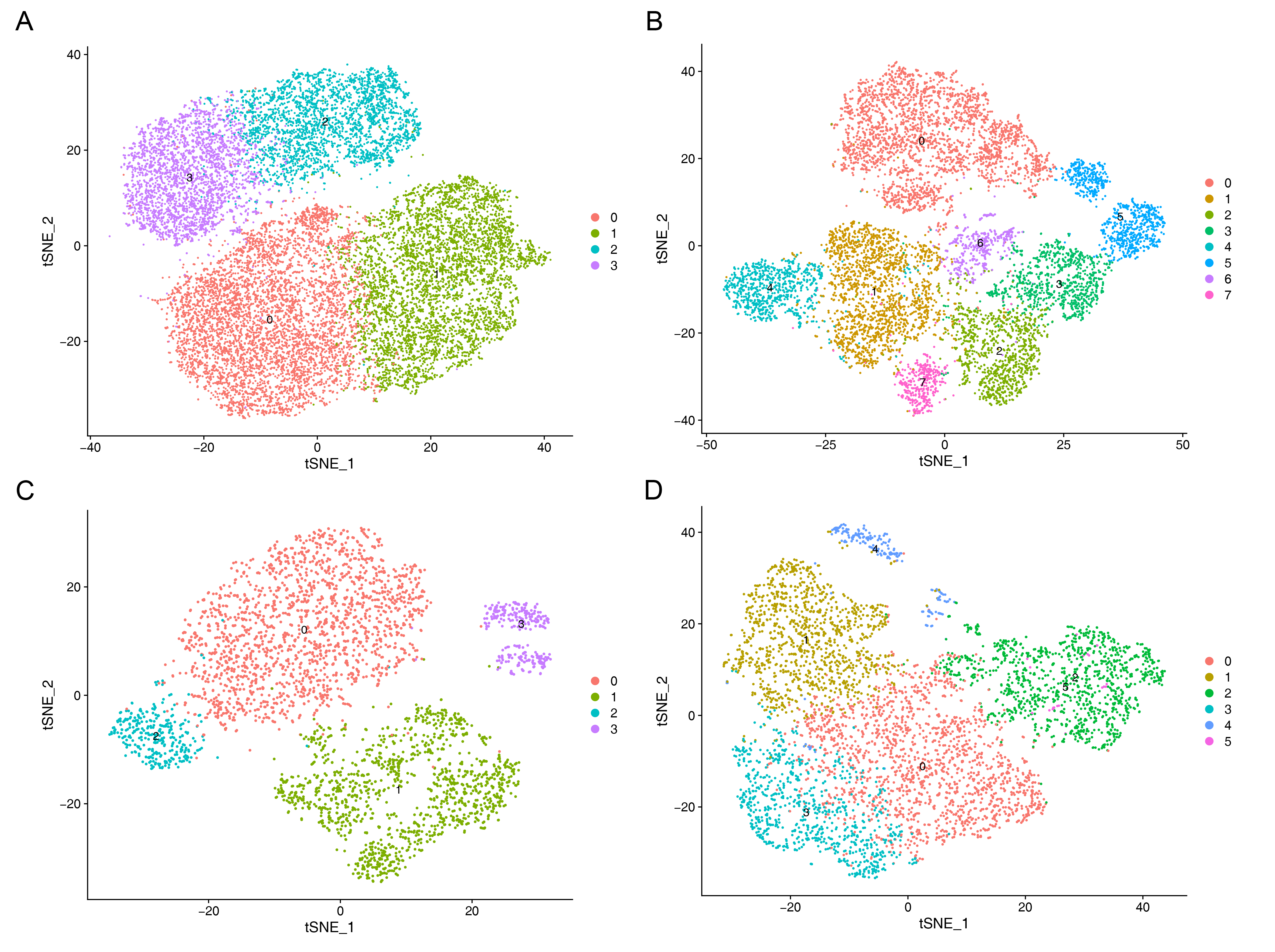

Supplement: SUPPLEMENTARY FIGURE 1 — Screen candidate key genes. (A) PPI network; (B) Cluster 1; (C) Cluster 2; (D) Cluster 3; (E,G) represent the error plots of Lasso cross-validation for AD and SD, respectively. A positive coefficient indicates that high expression of the gene increases the risk of the outcome, while a negative coefficient indicates that high expression of the gene reduces the risk of the outcome. Curves (in different colors): Changes in the regression coefficients of genes under different λ values; as λ increases, some curves gradually decrease to 0, indicating that the corresponding gene is eliminated by LASSO; (F,H) represent the gene coefficient plots of Lasso for AD and SD, respectively. Lines and labels in different colors: Each point represents a model corresponding to a λ value; colors and labels indicate the number of variables retained under that λ; (I) Venn diagram. [file Data_sheet_1.zip › Supplementary material/Supplementary Figure. 8.tif]

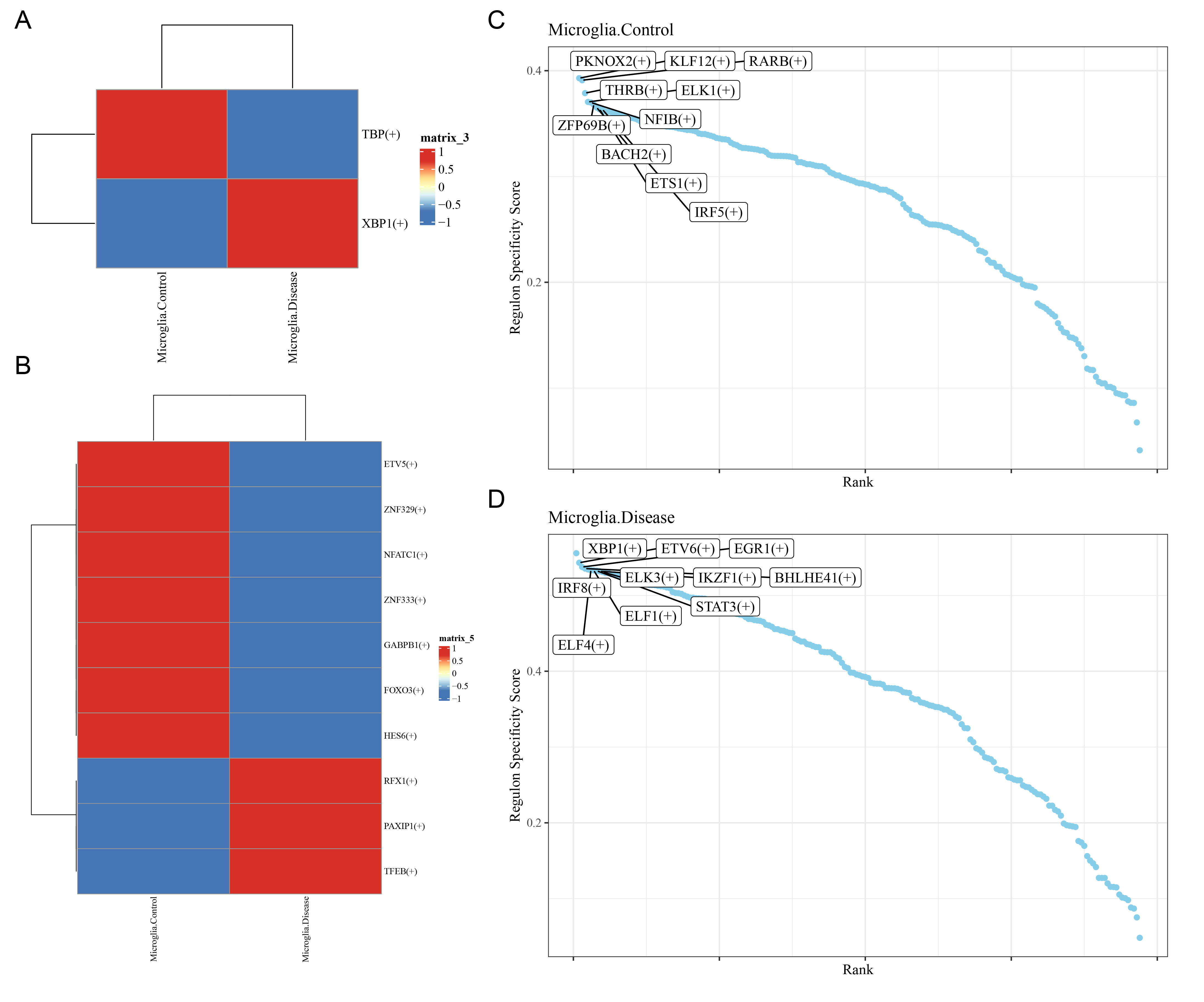

Supplement: SUPPLEMENTARY FIGURE 1 — Screen candidate key genes. (A) PPI network; (B) Cluster 1; (C) Cluster 2; (D) Cluster 3; (E,G) represent the error plots of Lasso cross-validation for AD and SD, respectively. A positive coefficient indicates that high expression of the gene increases the risk of the outcome, while a negative coefficient indicates that high expression of the gene reduces the risk of the outcome. Curves (in different colors): Changes in the regression coefficients of genes under different λ values; as λ increases, some curves gradually decrease to 0, indicating that the corresponding gene is eliminated by LASSO; (F,H) represent the gene coefficient plots of Lasso for AD and SD, respectively. Lines and labels in different colors: Each point represents a model corresponding to a λ value; colors and labels indicate the number of variables retained under that λ; (I) Venn diagram. [file Data_sheet_1.zip › Supplementary material/Supplementary Figure. 10.tif]

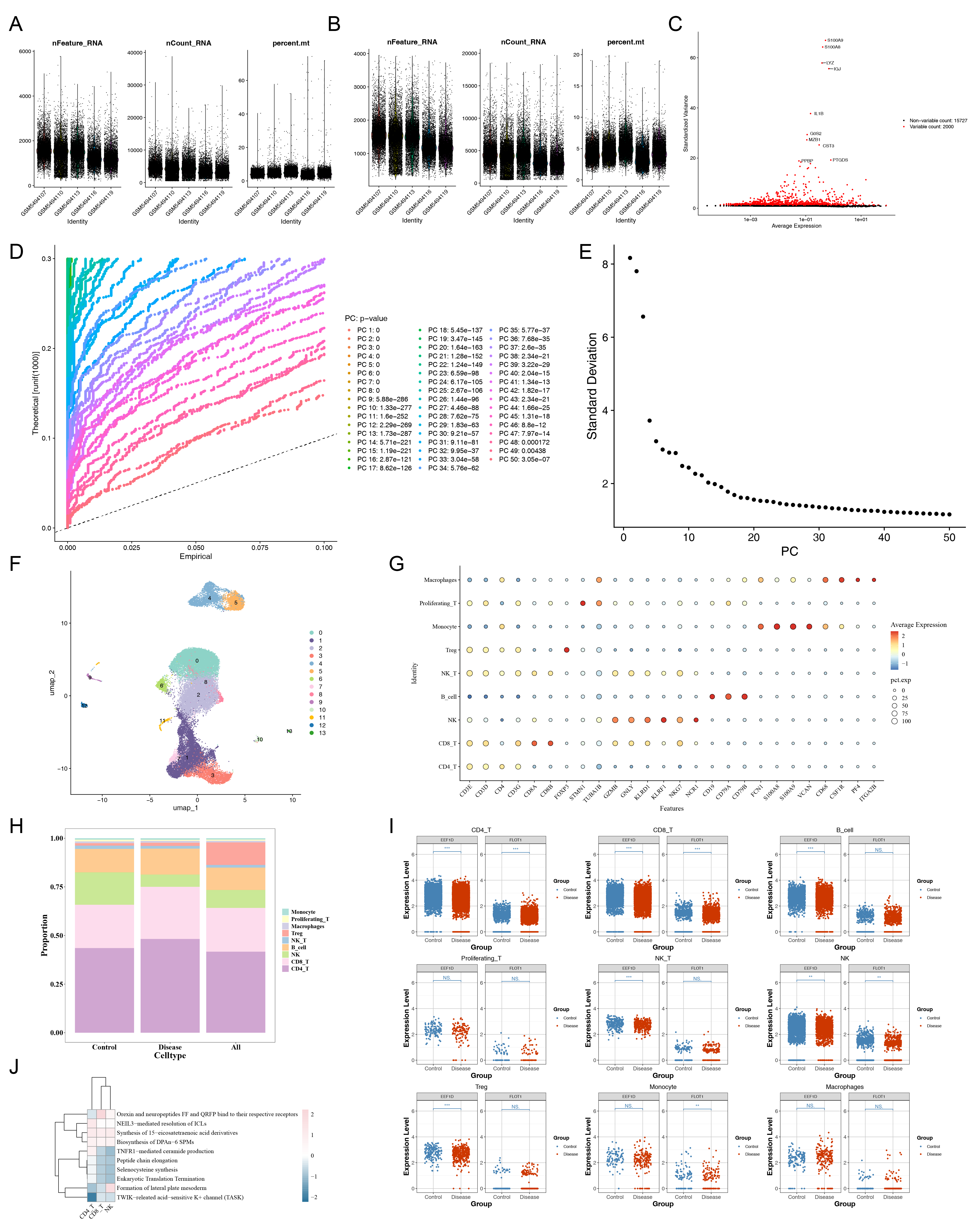

Supplement: SUPPLEMENTARY FIGURE 1 — Screen candidate key genes. (A) PPI network; (B) Cluster 1; (C) Cluster 2; (D) Cluster 3; (E,G) represent the error plots of Lasso cross-validation for AD and SD, respectively. A positive coefficient indicates that high expression of the gene increases the risk of the outcome, while a negative coefficient indicates that high expression of the gene reduces the risk of the outcome. Curves (in different colors): Changes in the regression coefficients of genes under different λ values; as λ increases, some curves gradually decrease to 0, indicating that the corresponding gene is eliminated by LASSO; (F,H) represent the gene coefficient plots of Lasso for AD and SD, respectively. Lines and labels in different colors: Each point represents a model corresponding to a λ value; colors and labels indicate the number of variables retained under that λ; (I) Venn diagram. [file Data_sheet_1.zip › Supplementary material/Supplementary Figure. 6.tif]

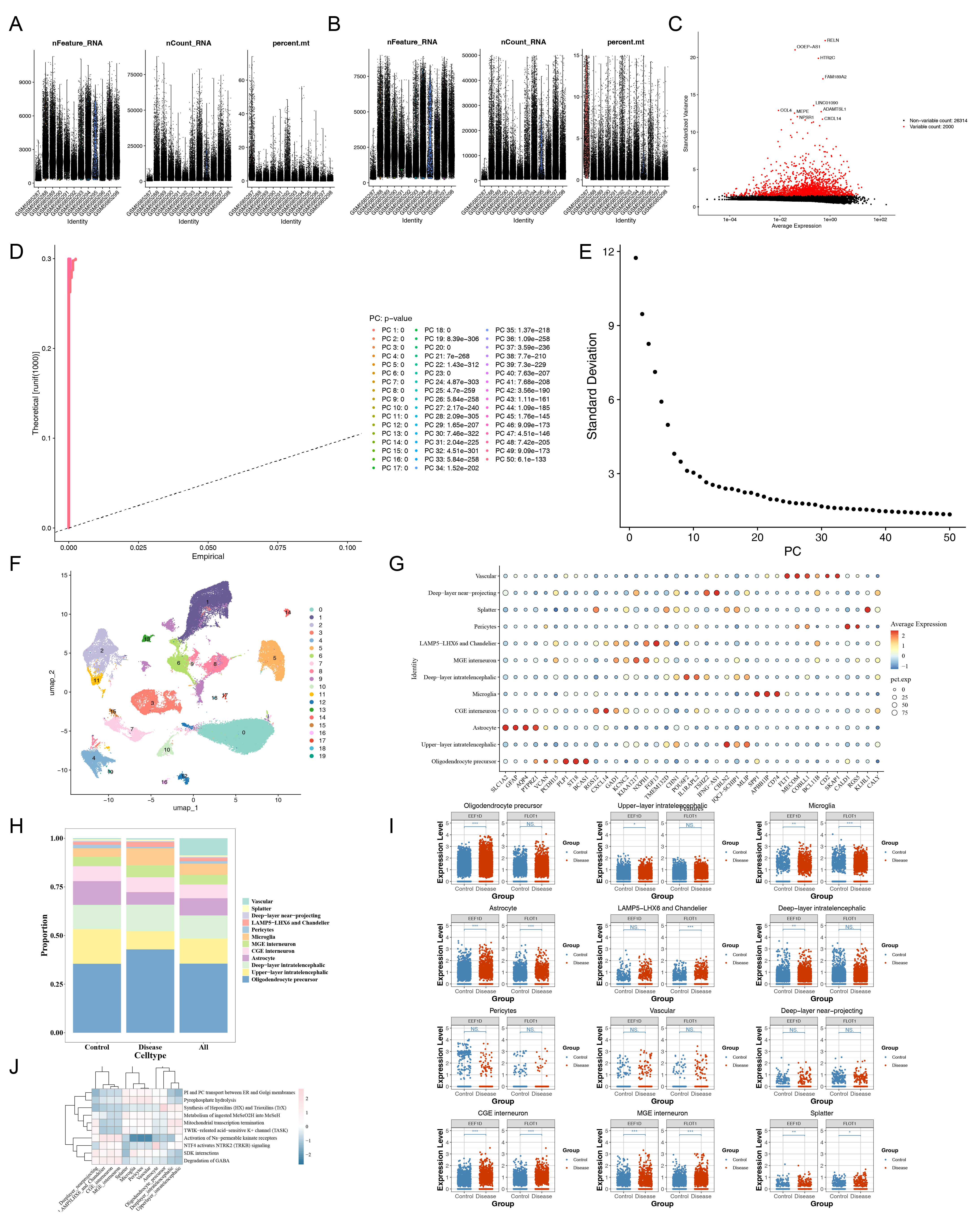

Supplement: SUPPLEMENTARY FIGURE 1 — Screen candidate key genes. (A) PPI network; (B) Cluster 1; (C) Cluster 2; (D) Cluster 3; (E,G) represent the error plots of Lasso cross-validation for AD and SD, respectively. A positive coefficient indicates that high expression of the gene increases the risk of the outcome, while a negative coefficient indicates that high expression of the gene reduces the risk of the outcome. Curves (in different colors): Changes in the regression coefficients of genes under different λ values; as λ increases, some curves gradually decrease to 0, indicating that the corresponding gene is eliminated by LASSO; (F,H) represent the gene coefficient plots of Lasso for AD and SD, respectively. Lines and labels in different colors: Each point represents a model corresponding to a λ value; colors and labels indicate the number of variables retained under that λ; (I) Venn diagram. [file Data_sheet_1.zip › Supplementary material/Supplementary Figure. 7.tif]

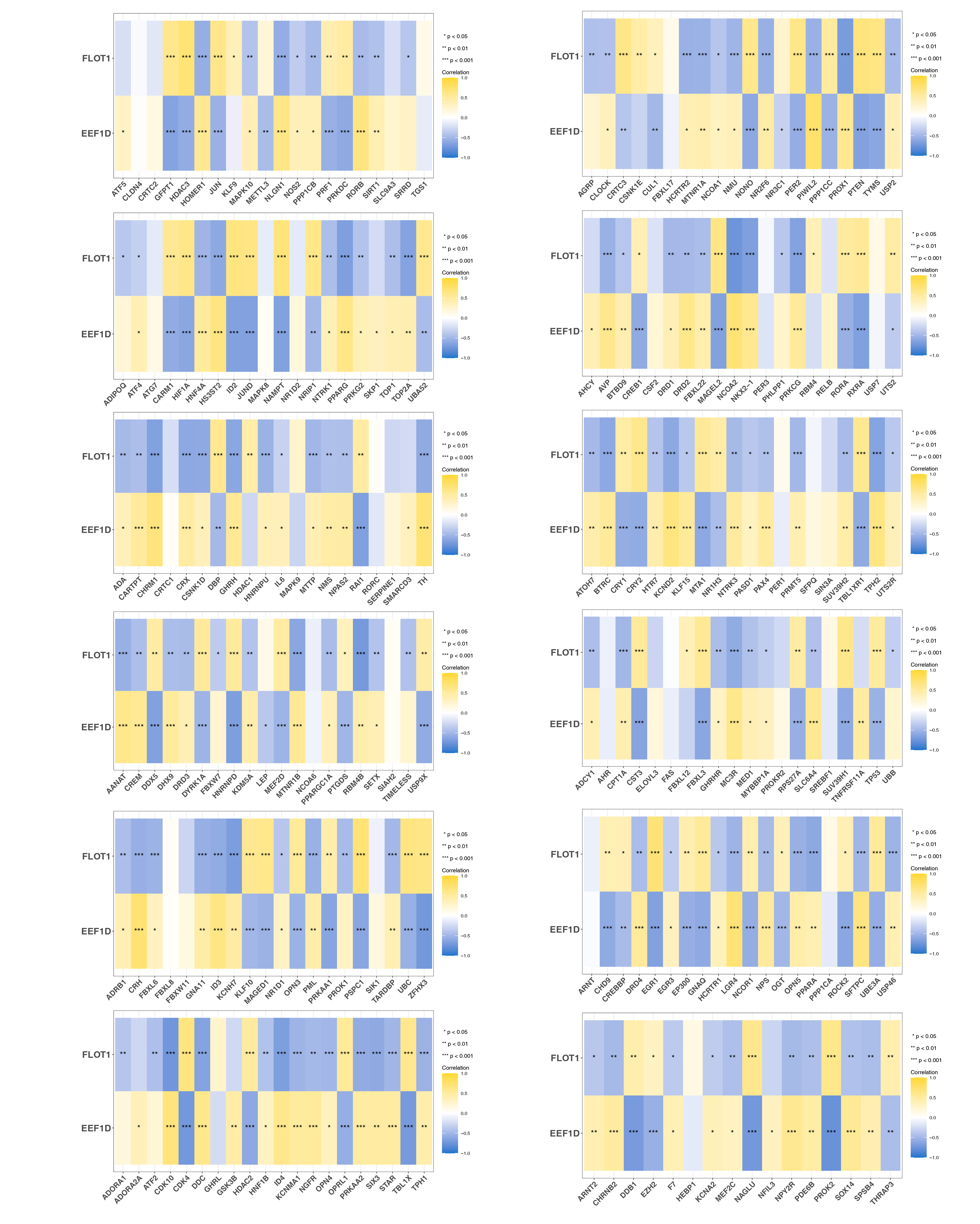

Supplement: SUPPLEMENTARY FIGURE 1 — Screen candidate key genes. (A) PPI network; (B) Cluster 1; (C) Cluster 2; (D) Cluster 3; (E,G) represent the error plots of Lasso cross-validation for AD and SD, respectively. A positive coefficient indicates that high expression of the gene increases the risk of the outcome, while a negative coefficient indicates that high expression of the gene reduces the risk of the outcome. Curves (in different colors): Changes in the regression coefficients of genes under different λ values; as λ increases, some curves gradually decrease to 0, indicating that the corresponding gene is eliminated by LASSO; (F,H) represent the gene coefficient plots of Lasso for AD and SD, respectively. Lines and labels in different colors: Each point represents a model corresponding to a λ value; colors and labels indicate the number of variables retained under that λ; (I) Venn diagram. [file Data_sheet_1.zip › Supplementary material/Supplementary Figure. 5.tif]

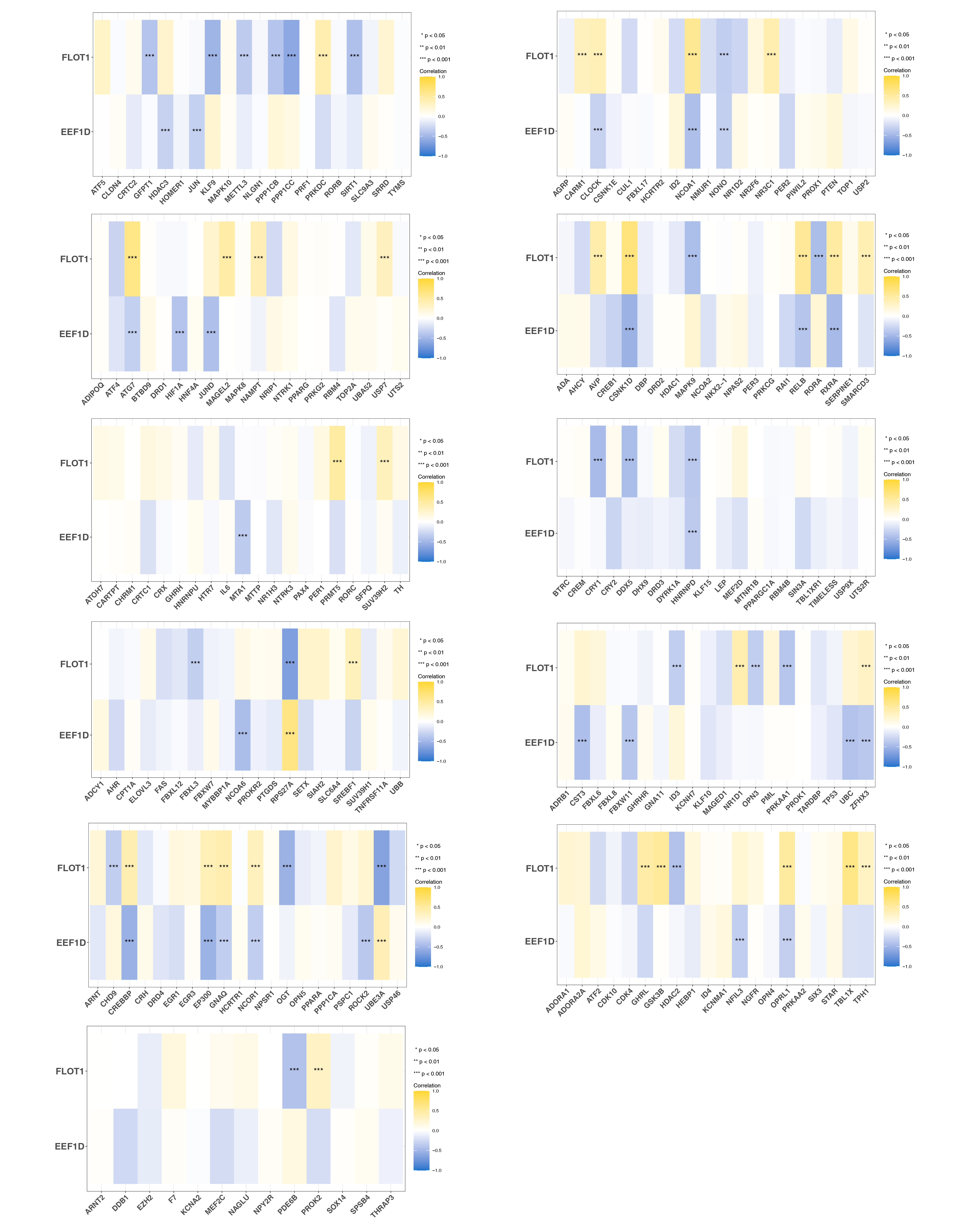

Supplement: SUPPLEMENTARY FIGURE 1 — Screen candidate key genes. (A) PPI network; (B) Cluster 1; (C) Cluster 2; (D) Cluster 3; (E,G) represent the error plots of Lasso cross-validation for AD and SD, respectively. A positive coefficient indicates that high expression of the gene increases the risk of the outcome, while a negative coefficient indicates that high expression of the gene reduces the risk of the outcome. Curves (in different colors): Changes in the regression coefficients of genes under different λ values; as λ increases, some curves gradually decrease to 0, indicating that the corresponding gene is eliminated by LASSO; (F,H) represent the gene coefficient plots of Lasso for AD and SD, respectively. Lines and labels in different colors: Each point represents a model corresponding to a λ value; colors and labels indicate the number of variables retained under that λ; (I) Venn diagram. [file Data_sheet_1.zip › Supplementary material/Supplementary Figure. 4.tif]

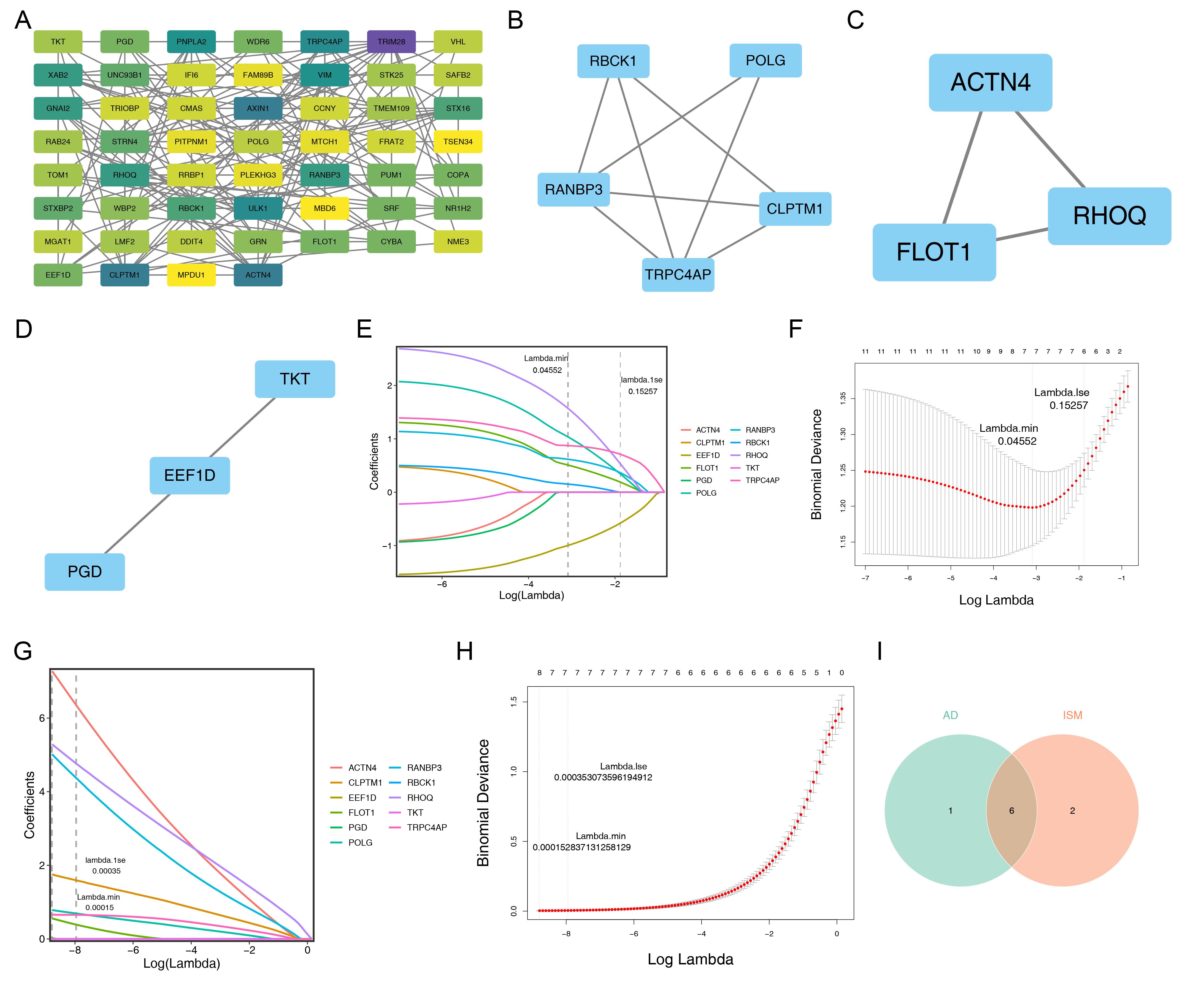

Supplement: SUPPLEMENTARY FIGURE 1 — Screen candidate key genes. (A) PPI network; (B) Cluster 1; (C) Cluster 2; (D) Cluster 3; (E,G) represent the error plots of Lasso cross-validation for AD and SD, respectively. A positive coefficient indicates that high expression of the gene increases the risk of the outcome, while a negative coefficient indicates that high expression of the gene reduces the risk of the outcome. Curves (in different colors): Changes in the regression coefficients of genes under different λ values; as λ increases, some curves gradually decrease to 0, indicating that the corresponding gene is eliminated by LASSO; (F,H) represent the gene coefficient plots of Lasso for AD and SD, respectively. Lines and labels in different colors: Each point represents a model corresponding to a λ value; colors and labels indicate the number of variables retained under that λ; (I) Venn diagram. [file Data_sheet_1.zip › Supplementary material/Supplementary Figure. 1.tif]

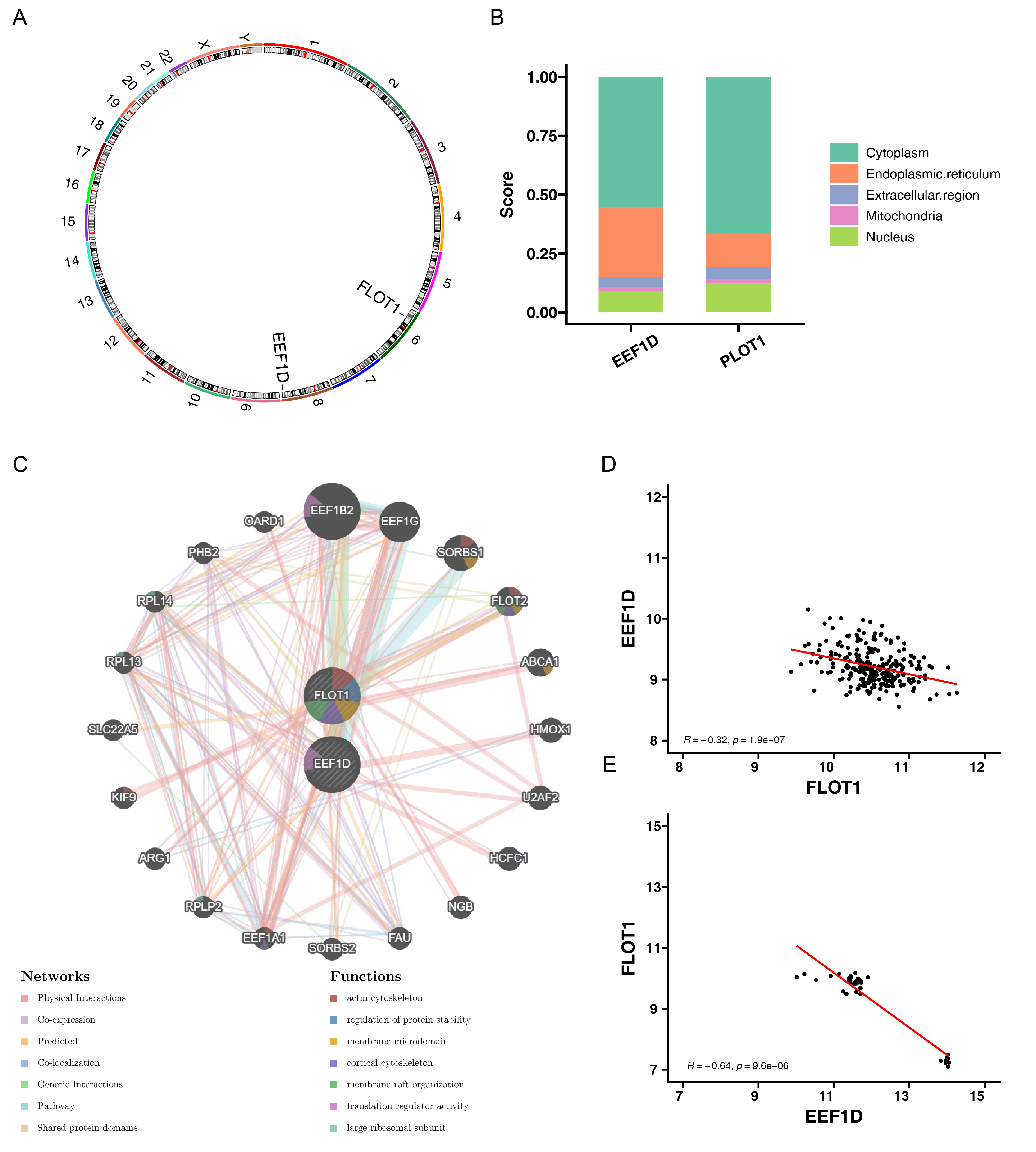

Supplement: SUPPLEMENTARY FIGURE 1 — Screen candidate key genes. (A) PPI network; (B) Cluster 1; (C) Cluster 2; (D) Cluster 3; (E,G) represent the error plots of Lasso cross-validation for AD and SD, respectively. A positive coefficient indicates that high expression of the gene increases the risk of the outcome, while a negative coefficient indicates that high expression of the gene reduces the risk of the outcome. Curves (in different colors): Changes in the regression coefficients of genes under different λ values; as λ increases, some curves gradually decrease to 0, indicating that the corresponding gene is eliminated by LASSO; (F,H) represent the gene coefficient plots of Lasso for AD and SD, respectively. Lines and labels in different colors: Each point represents a model corresponding to a λ value; colors and labels indicate the number of variables retained under that λ; (I) Venn diagram. [file Data_sheet_1.zip › Supplementary material/Supplementary Figure. 3.tif]

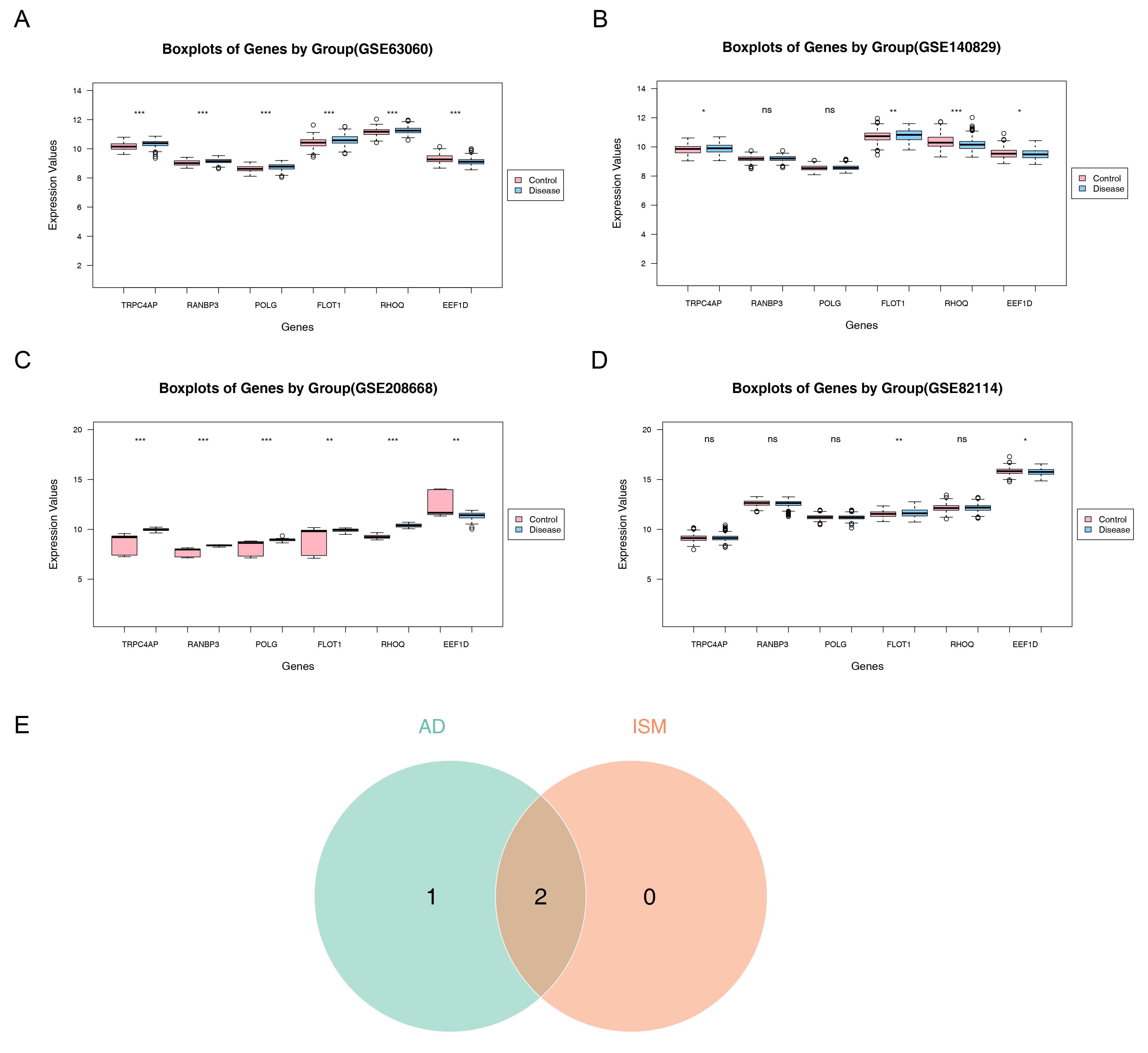

Supplement: SUPPLEMENTARY FIGURE 1 — Screen candidate key genes. (A) PPI network; (B) Cluster 1; (C) Cluster 2; (D) Cluster 3; (E,G) represent the error plots of Lasso cross-validation for AD and SD, respectively. A positive coefficient indicates that high expression of the gene increases the risk of the outcome, while a negative coefficient indicates that high expression of the gene reduces the risk of the outcome. Curves (in different colors): Changes in the regression coefficients of genes under different λ values; as λ increases, some curves gradually decrease to 0, indicating that the corresponding gene is eliminated by LASSO; (F,H) represent the gene coefficient plots of Lasso for AD and SD, respectively. Lines and labels in different colors: Each point represents a model corresponding to a λ value; colors and labels indicate the number of variables retained under that λ; (I) Venn diagram. [file Data_sheet_1.zip › Supplementary material/Supplementary Figure. 2.tif]
